# HDAC6 Degradation Inhibits the Growth of High-Grade Serous Ovarian Cancer Cells

**DOI:** 10.3390/cancers12123734

**Published:** 2020-12-11

**Authors:** Ahlam Ali, Fengyu Zhang, Aaron Maguire, Tara Byrne, Karolina Weiner-Gorzel, Stephen Bridgett, Sharon O’Toole, John O’Leary, Caitlin Beggan, Patricia Fitzpatrick, Amanda McCann, Fiona Furlong

**Affiliations:** 1 Patrick G Johnston Centre for Cancer Research, Queen’s University, Belfast BT97BL, UK; a.ali@qub.ac.uk; 2 School of Pharmacy, Queen’s University, Belfast BT97BL, UK; fzhang04@qub.ac.uk (F.Z.); amaguire539@qub.ac.uk (A.M.); tbyrne03@qub.ac.uk (T.B.); S.Bridgett@qub.ac.uk (S.B.); 3 UCD Conway Institute of Biomolecular and Biomedical Research, UCD School of Medicine, College of Health and Agricultural Sciences (CHAS), University College Dublin (UCD), Belfield, Dublin 4, Ireland; karolinaweinergorzel@gmail.com (K.W.-G.); amanda.mccann@ucd.ie (A.M.); 4 Department of Histopathology, Trinity College Dublin and Trinity St James’s Cancer Institute, St. James’s Hospital, Dublin 8, Ireland; shotoole@tcd.ie; 5 Emer Casey Molecular Pathology Research Laboratory, Coombe Women’s & Infants University Hospital, Dublin D08, Ireland; 6 Department of Obstetrics & Gynaecology, Trinity College Dublin and Trinity St James’s Cancer Institute, Trinity Centre for Health Sciences, St James’s Hospital, Dublin 2, Ireland; olearyjj@tcd.ie; 7 Department of Pathology, Royal College of Surgeons in Ireland (RCSI), Beaumont Hospital, Dublin 9, Ireland; caitlin.beggan@stgeorges.nhs.uk; 8 School of Public Health, Physiotherapy & Sports Science, College of Health and Agricultural Sciences, University College Dublin (UCD), Belfield, Dublin 4, Ireland; patricia.fitzpatrick@ucd.ie

**Keywords:** HDAC6, HGSOCs, ACY-1215, paclitaxel, cisplatin, survival

## Abstract

**Simple Summary:**

The objective of this study was, firstly, to investigate the relationship between Histone deacetylase 6 (HDAC6) expression and survival in patients with ovarian cancer and, secondly, to test the effects of histone deacetylase 6 (HDAC6) inhibition on ovarian cancer cells in vitro. A meta-analysis of the correlation between HDAC6 gene expression and survival was performed on 3573 ovarian tumors from 19 datasets showed that high HDAC6 gene expression was associated with a decreased risk of death. Knockdown of HDAC6 gene expression with small interfering RNA (siRNA) and protein expression with a HDAC6 targeting protein degrader decreased ovarian cell proliferation, migration, and viability. Conversely, the selective inhibition of HDAC6 catalytic activity did not produce a robust inhibition of HDAC6 protein function. In summary, we demonstrated, for the first time, that HDAC6 over-expression in ovarian cancers is a favorable prognostic marker. We provide evidence to suggest that inhibition of HDAC6 catalytic activity has limited efficacy as a monotherapy in ovarian cancers.

**Abstract:**

Histone deacetylase 6 (HDAC6) is a unique histone deacetylating enzyme that resides in the cell cytoplasm and is linked to the modulation of several key cancer related responses, including cell proliferation and migration. The promising anti-cancer response of the first-generation HDAC6 catalytic inhibitors continues to be assessed in clinical trials, although its role in high grade serous ovarian cancer is unclear. This study investigated HDAC6 tumor expression by immunohistochemistry in high-grade serous ovarian cancer (HGSOC) tissue samples and a meta-analysis of HDAC6 gene expression in ovarian cancer from publicly available data. The pharmacological activity of HDAC6 inhibition was assessed in a patient-derived model of HGSOC. HDAC6 was found to be highly expressed in HGSOC tissue samples and in the patient-derived HGSOC cell lines where higher HDAC6 protein and gene expression was associated with a decreased risk of death (hazard ratio (HR) 0.38, (95% confidence interval (CI), 0.16–0.88; *p* = 0.02); HR = 0.88 (95% CI, 0.78–0.99; *p* = 0.04)). Similarly, the multivariate analysis of HDAC6 protein expression, adjusting for stage, grade, and cytoreduction/cytoreductive surgery was associated with a decreased risk of death (HR = 0.19 (95% CI, 0.06–0.55); *p* = 0.002). Knock-down of HDAC6 gene expression with siRNA and protein expression with a HDAC6 targeting protein degrader decreased HGSOC cell proliferation, migration, and viability. Conversely, the selective inhibition of HDAC6 with the catalytic domain inhibitor, Ricolinostat (ACY-1215), inhibited HDAC6 deacetylation of α-tubulin, resulting in a sustained accumulation of acetylated α-tubulin up to 24 h in HGSOC cells, did not produce a robust inhibition of HDAC6 protein function. Inhibition of HGSOC cell proliferation by ACY-1215 was only achieved with significantly higher and non-selective doses of ACY-1215. In summary, we demonstrated, for the first time, that HDAC6 over-expression in HGSOC and all ovarian cancers is a favorable prognostic marker. We provide evidence to suggest that inhibition of HDAC6 catalytic activity with first generation HDAC6 inhibitors has limited efficacy as a monotherapy in HGSOC.

## 1. Introduction

Ovarian cancer is the leading cause of death from a gynecological cancer and ranks fourth as the leading cause of death from a fatal disease in women, which led to ~14,170 deaths in 2019 [1]. High-grade serous ovarian cancer (HGSOC) is the most common and aggressive type of epithelial ovarian cancer [1]. Unfortunately, most patients are diagnosed with a late stage disease and the five-year survival for these patients has remained static at around 30% [2]. The standard of care for the majority of HGSOC patients is primary cytoreductive (debulking) surgery, followed by adjuvant platinum and paclitaxel chemotherapy [3]. Progress in the treatment of ovarian cancer has seen the introduction of targeted therapies, such as bevacizumab and ploy(adenosine diphosphate-ribose) polymerase (PARP) inhibitors, and a plethora of immunotherapy trials [4]. However, long-term benefit for most patients with HGSOC has not yet been achieved and efforts to identify early detection biomarkers, targeted therapies and associated stratification biomarkers continues to be a major unmet clinical need for these patients.

Histone deacetylase inhibitors (HDACi) are known to modify the acetylation status of many histone and non-histone proteins that have crucial roles in numerous biological processes, such as cell proliferation, differentiation, angiogenesis, apoptosis, autophagy, and tumor immunity, consequently exhibiting antitumor activity [5]. Currently, four drugs, vorinostat, also known as suberoylanilide hydroxamic acid (SAHA): a potent HDAC inhibitor [6]; romidepsin (FK-228) [7]; belinostat (PXD-101) [8]; and panobinostat (LBH-589) [9], have been granted Federal Drug Adiministartion (FDA) approval for cancer treatment, and several HDACis are currently in various phases of clinical trials, either as mono-therapy and/or in combination with existing/novel anticancer agents [10]. Although HDACi has demonstrated significant anti-tumor activity, the dose-limiting side effects associated with their use severely prevent their wide scale utilization in the clinic. However, unlike the inhibition of other HDACs, the inhibition of Histone deacetylase 6 (HDAC6) is not believed to be associated with such severe toxicities [11]. Mice lacking HDAC6 develop normally with normal major organ function [12], and, over the last decade, major efforts have led to the development of selective HDAC6 inhibitors. Ricolinostat (ACY-1215) is currently undergoing phase I and II clinical trials in various diseases, including breast cancer [13].

HDAC6 is a cytoplasmic enzyme that is highly expressed in breast cancer [14], advanced primary acute myeloid leukemia (AML) [15], primary oral squamous [16], and laryngeal squamous cell carcinoma [17]. High expression of HDAC6 was proposed to be linked with tumor aggressiveness. In prostate cancer, HDAC6 expression levels was not associated with disease outcomes [18], while increased HDAC6 expression was shown to be associated with a more advanced stage in oral squamous cancers [16] and a poorer prognosis in high grade gliomas [19]. Improved survival was shown to be associated with moderate to strong HDAC6 immunohistochemistry (IHC) expression in diffuse large B-cell [20], and breast cancer [21]. In ovarian cancer, HDAC6 expression levels were shown to be higher in low-grade and high-grade cancers compared to benign lesions [22]. In addition, Yano et al., 2018 [23], demonstrated a relationship between high HDAC6 nuclear overexpression and poor prognosis in ovarian cancer, especially in surgical suboptimal cases.

HDAC6 modulates the stress response, oncogenesis, cell motility, and many other cancer-related signaling networks in ovarian cancer cells [10,24], and its inhibition significantly improved the survival of mice bearing AT-Rich Interaction Domain 1A (ARID1A)-mutated ovarian tumors, correlating with suppressed tumor growth and dissemination of ARID1A-mutated, but not wild-type, tumors [25]. In addition, targeting the ubiquitin-proteasome system (UPS), with Bortezomib, combined with HDAC6 inhibition was shown to (i) successfully kill ovarian cancer cells in vitro and (ii) reduce ovarian cancer cell spreading and migration as a result of the deacetylation of α-tubulin by HDAC6 [22]. Resistance to paclitaxel, a first line therapy in the treatment for ovarian cancer, was reversed with HDAC6 inhibition [26,27] in which combination treatment of HDAC6i and Paclitaxel resulted in the synergistic killing of resistant ovarian cancer cells in vitro [28] and in vivo [29]. However, it is now well recognized that many of the earlier studies of HDAC6 inhibition were performed with non-selective HDAC6 inhibitors or at non-selective drug concentrations [30]. Moreover, Lin et al. used CRISPR-Cas9 to study the knock-out of several cancer targets suggested that HDAC6 had no anti-cancer activity in several cancer cell lines, although ovarian cancer cells were not tested in this study [31]. Therefore, this study investigated the potential anticancer activity of selective HDAC6 inhibition in a patient-derived cell model of HGSOC by comparing HDAC6 knockdown with the pharmacological inhibition of HDAC6.

## 2. Results

### 2.1. Low HDAC6 Expression is Associated with Poorer Survival in Ovarian Cancer

**Figure 1:**
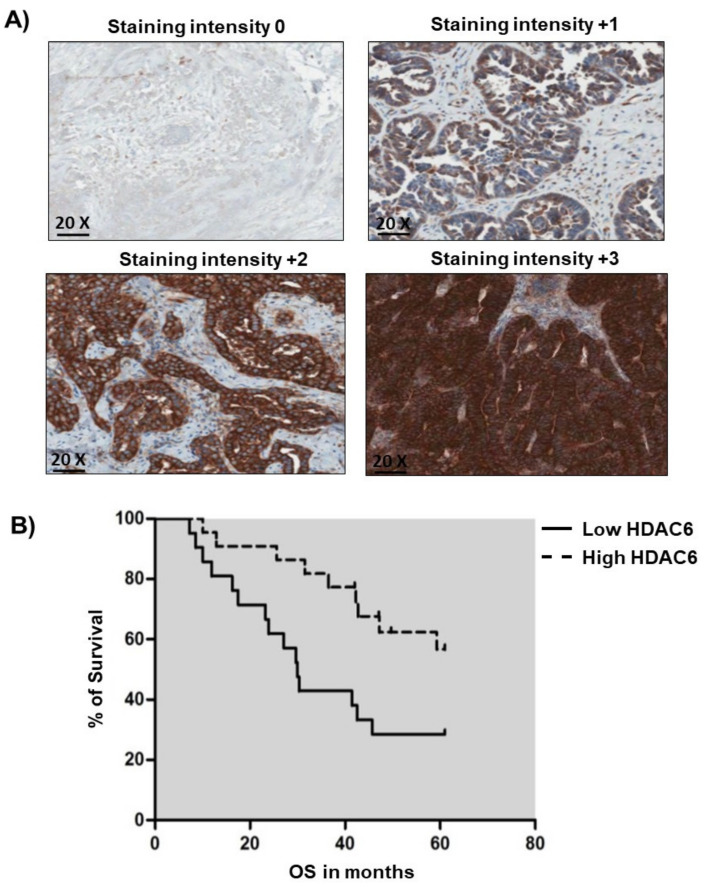
High Histone deacetylase 6 (HDAC6) protein expression is associated with a reduced risk of death. (**A**) Low HDAC6 staining intensity associates with poor OS for patients with High-grade serous ovarian cancer (HGSOC). Full-face formalin-fixed paraffin embedded (FFPE) sections demonstrating immunohistochemistry (IHC) staining intensity for HDAC6: negative 0, low +1, intermediate +2, high +3 taken at 20× magnification. (**B**) Univariate Cox’s regression hazard analysis. Correlation of low (staining intensity negative, low +1 and intermediate +2) HDAC6 IHC staining intensity expression with OS in patients with HGSOC (HR 0.38, (95% CI, 0.16–0.88; *p* = 0.02). Multivariate Cox’s regression hazard analysis (adjusted for stage, tumor grade and optimal surgical debulking < 1 cm) showing a significant correlation between low HDAC6 IHC staining intensity and OS (HR = 0.19 (95% CI, 0.06–0.549); *p* = 0.002).

Ovarian cancer represents a subset of pathogenically, histological, and molecularly separate diseases consisting of five major subtypes (HGSOC, low-grade serous carcinoma, endometrioid carcinoma, clear cell carcinoma, and mucinous carcinoma) which should be assessed individually [32]. While HDAC6 can be over-expressed in ovarian cancer cells, few studies have assessed the contribution of HDAC6 expression to HGSOC prognosis. Immunohistochemistry of 46 formalin-fixed paraffin embedded (FFPE) sections of HGSOC (Table 1) demonstrated that HDAC6 predominantly localized to the cytoplasm at varying degrees of intensity (Figure 1A). The resultant Kaplan-Meier survival curve for HDAC6 staining intensity and overall survival (OS) also demonstrated that low HDAC6 was significantly associated with poorer OS in a univariate analysis (HR 0.38, (95% CI, 0.16–0.88; *p* = 0.02) (Figure 1B). In a multivariate Cox regression analysis adjusting for stage, grade, and cytoreduction/cytoreductive surgery (<1 cm), the association between low HDAC6 levels and OS remained independently statistically significant, with a further decreased HR of 0.19 ((95% CI, 0.06–0.549); *p* = 0.002)) (Figure S1C). Moreover, a pooled hazard ratio (HR) for OS of HDAC6 mRNA levels in patients with low-expressing HDAC6 tumors compared to those with high expressing HDAC6 tumors was 0.88 (95% CI, 0.78–0.99; *p* = 0.04), indicating an association between low HDAC6 mRNA expression and a decreased risk of survival (Table S1). Therefore, we present the first study to demonstrate that low HDAC6 expression is significantly associated with poorer overall survival by a univariate and multivariate analysis of IHC in HGSOC specimens, as well as a meta-analysis of mRNA expression in ovarian cancer.

### 2.2. Characterisation of a Patient-Derived Cell Model of HGSOC

**Figure 2:**
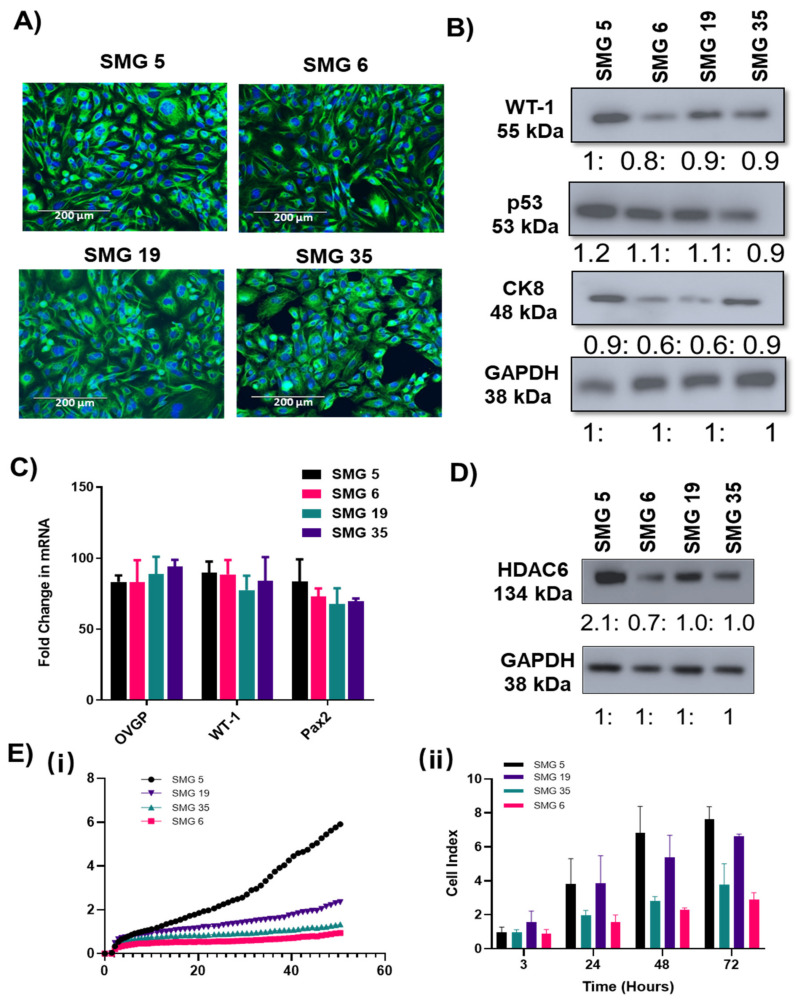
Molecular profiling of new established SMG cells: (**A**) Immunocytochemical analysis for the expression of ovarian epithelial biomarker protein CK8 in SMG ovarian cancer cell lines. (Scale bar = 200 µm) (*n* = 3). (**B**) Western blotting showing expression profiles of ovarian markers including wild type (WT)-1, p53, and CK8. (*n* = 3 ± SEM). Uncropped Western Blots are available in Figure S6. (**C**) qRT-PCR showing gene expression profiles of ovarian markers including WT-1, OVGP, and Pax8 in SMG ovarian cancer cell lines. Delta CT values were normalized to the housekeeping gene β-actin. *n* = 3 ± SEM. (**D**) Representative images of basal levels of HDAC6 in SMG cells measured by western blot analysis. GAPDH was used as a loading control for both blots. *n* = 3 ± SEM. Uncropped Western Blots are available in Figure S7. (**E**)/(**i**) Representative graphs from the real time xCELLigence proliferation system.(**E**), and average data at selected time points(**ii**). Cell index values taken from the xCELLigence system monitoring the cells at time intervals, observing the rate of adhesion/spreading (3 h) and proliferation (6–70 h). Wells were analyzed in duplicate, and two biological replicates were performed to validate results, n = 2 ± SEM.

The most regularly used cell line models for ovarian cancer and implicitly for the most prevalent subtype HGSOC are SKOV-3, A2780, OVCAR-3, CAOV3, and IGROV1,;however, their histo-pathological origin is partly unclear, and the need for well-characterized cell lines as models for the respective subtypes of ovarian cancer has been repeatedly expressed [33]. To address this, a panel of SV-40 transformed patient-derived cells (SMG cells) were used in this study. The cells were established from ascites fluid that represent different stages of HGSOC. Starting from early passages, SMG cells have shown relatively low morphological heterogeneity. The cells are medium sized, with epithelial morphology, as shown by immunohistochemistry staining of an ovarian cancer epithelial marker, cytokeratin 8 (CK8) (Figure 2A). HGSOCs are typically associated with high frequency of p53 mutations, as well as elevated (Wilms tumor 1 WT1 in > 80% of the cases [31,34]. In addition, most inclusion cysts and fallopian tube epithelium are composed of CK8/paired box gene 8 (PAX8) and oviductal glycoprotein 1(OVGP1) positive cells. This distinctive profile helps distinguish ovarian epithelium from other tissues [35,36]. Consistent with this, validation studies showed by immunoblotting and with RT-PCR (Figure 2B,C) demonstrates the overexpression of these markers in the SMG cell lines, reassuring that the cell lines used in this study were HGSOC phenotype.

Although HDAC6 becomes over-expressed in cancer [22], there are too few studies to directly evaluate this in ovarian cancer and no reported studies profiling HDAC6 expression comparing the expression in normal fallopian tubes with HGSOC precancerous lesions and to HGSOC. However, among the cell lines tested in this study (Figure 2D and Figure S1A), all cells expressed high levels of HDAC6 and the metastatic ovarian carcinoma cell line, SMG 5 and SMG 19, demonstrated very strong HDAC6 expression, which also correlated with greater cell proliferation compared with the low HDAC6 expressing SMG 6 and 35 cells (Figure 2E). Previous literature highlights that HDAC6 has a prominent role in cellular proliferation [17,37].

As part of the characterization of the SMG cells, drug responses of the corresponding cell lines were tested with paclitaxel and cisplatin, which are the two most commonly used first-line drugs for the treatment of ovarian cancer [32]. Both chemotherapeutics showed similar responses in 3 out of 4 cells lines tested (SMG 6, SMG 19, SMG 35) (Figure S2A,B). However, SMG 5 required higher cytotoxic doses of both paclitaxel and cisplatin (IC50 = 25 nM for paclitaxel and 95 nM for cisplatin compared to SMG 6; IC50 = 10 nM for paclitaxel and 14 nM for cisplatin). All cell lines are sensitive to both chemotherapies and the higher doses required to treat the SMG5 cells could reflect the higher proliferative capacity of these cells (Figure 2E).

### 2.3. HDAC6 Knockdown Significantly Inhibited Cell Proliferation and Migration HGSOC

**Figure 3:**
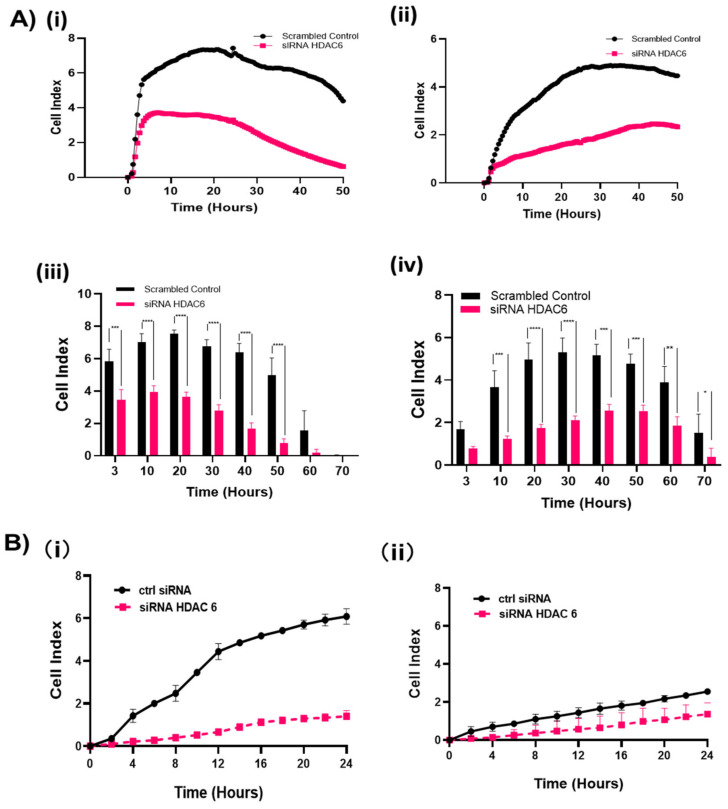
siRNA-mediated knockdown of HDAC6 caused lower cell spreading, cell proliferation, and cell migration. Cells were transfected with HDAC6 siRNA or negative scrambled control for 48 h followed by continuous measure of cell index by xCELLigence system. (**A**)/(**i**),(**ii**) Representative graphs from the real time xCELLigence proliferation system comparing growth profiles of scrambled siRNA treated SMG cells and HDAC6 transfected SMG cells. (**A**)/(**iii**),(**iv**) Cell index values taken from the xCELLigence system monitoring the cells at time intervals, observing the rate of adhesion/spreading (3 h) and proliferation (6–70 h). Wells were analyzed in duplicate, and two biological replicates were performed to validate results, *n* = 2. Experiments were analyzed by a two-way ANOVA. * *p* < 0.05, ** *p* < 0.01, *** *p* < 0.001, **** *p* < 0.0001. (**A**)/(**i**),(**ii**) SMG5, (**A**)/(**iii**),(**iv**) SMG6. (**B**) Migration assay with SMG5 cells (**B**)/(**i**) and SMG6 cells (**B**)/(**ii**) measured continuously as cell index using the migration xCELLigence system following siRNA transfection. Wells were analyzed in duplicate and two biological replicates were performed to validate results.

To measure the effects of HDAC6 on cell proliferation, two independent HDAC6 targeting siRNA oligos were used to knockdown HDAC6 and cell proliferation was monitored in real time for 96 h in the SMG 5 and SMG 6 cells. Knockdown was confirmed by immunoblotting (Figure S2A). Following incubation with HDAC6 targeting or control siRNA for 48 h, cells were re-plated on the xCELLigence plate (ACEC bioscience, Inc., Santa Clara, CA, USA) and continuously monitored for cell spreading, cell proliferation, or cell migration. HDAC6 knockdown significantly (*p* < 0.002) impaired cell proliferation in both the SMG cell lines (Figure 3A). Cell migration was compromised in both cell lines by HDAC6 knockdown, in which loss of HDAC6 more strongly affected SMG5 cell line migration (Figure 3B and Figure S3).

### 2.4. Pharmacological Inhibition of HDAC6 Slows the Growth of HGSOC Cells

**Figure 4:**
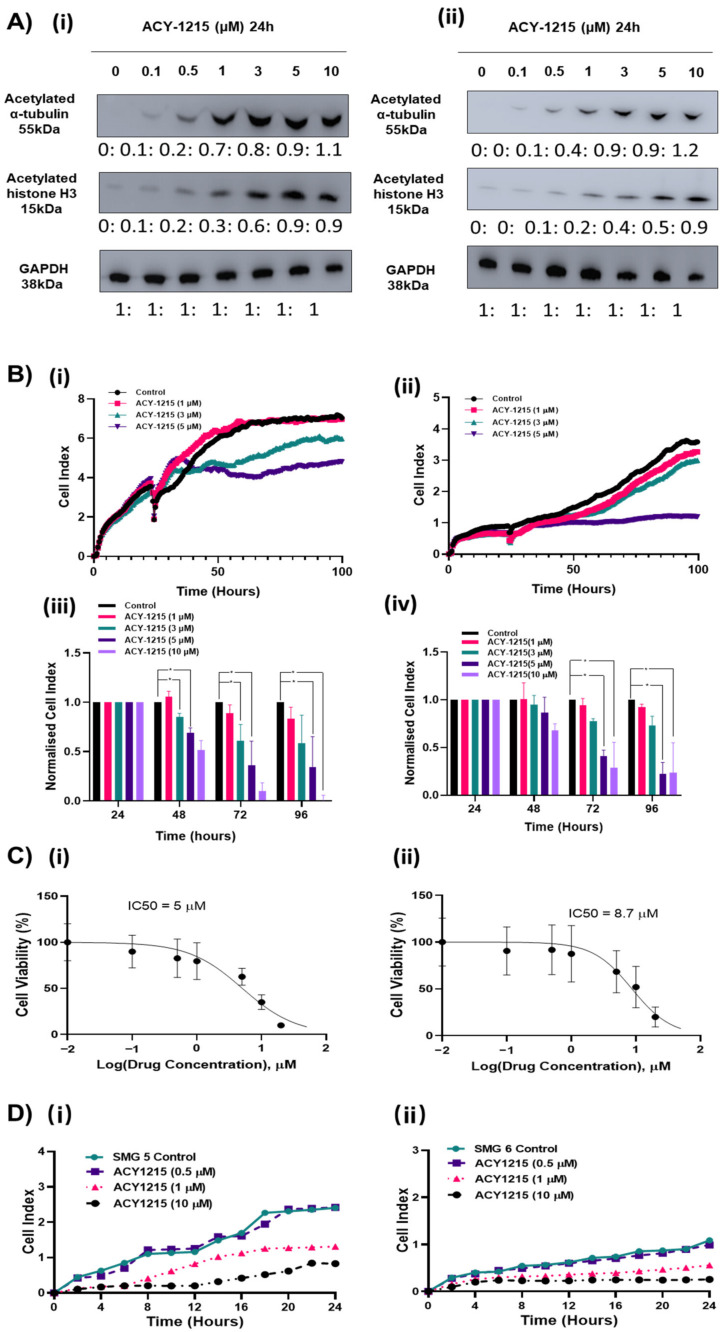
Low concentrations of the HDAC6 inhibitor ACY-1215 increased acetylated α-tubulin without affecting cell proliferation and viability. (**A**) SMG5 (**i**) and SMG6 (**ii**) cells treated with 1–10 μM of ACY-1215 for 24 h and α-tubulin and H3 levels were measured by western blot analysis. GAPDH was used as a loading control (*n* = 3). Uncropped Western Blots are available in Figures S8 and S9. (**B**) (**i)**,(**ii**) Representative graphs from the real time xCELLigence proliferation system comparing growth profiles of SMG cells after treated with 1–10 μM ACY-1215. (**iii**),(**iv**) Cell index values were normalized to both 24 h and control, observing the rate of proliferation (24–96 h). Wells were analyzed in duplicate, and three biological replicates were performed to validate results. Experiments were analyzed by a two-way ANOVA. * *p* < 0.05. Error bars represent SEM. (**i)**,(**iii**) SMG5, (**ii)**,(**iv**) SMG6. (**C**) SMG5 (**C**): (**i**) and SMG6 (**ii**) cells treated with 0–50 μM ACY-1215 and cell viability was measured using 3-(4,5-dimethylthiazol-2-yl)-2,5-diphenyl-2H-tetrazolium bromide-MTT assay (*n* = 3). (**D**) Representative graphs from the real time xCELLigence migration system comparing growth profiles of SMG5 (**i**) and SMG6 (**ii**) cells following pre-treatment with 1 or 10 μM ACY-1215. Wells were analyzed in duplicate, and three biological replicates were performed to validate results.

**Figure 5:**
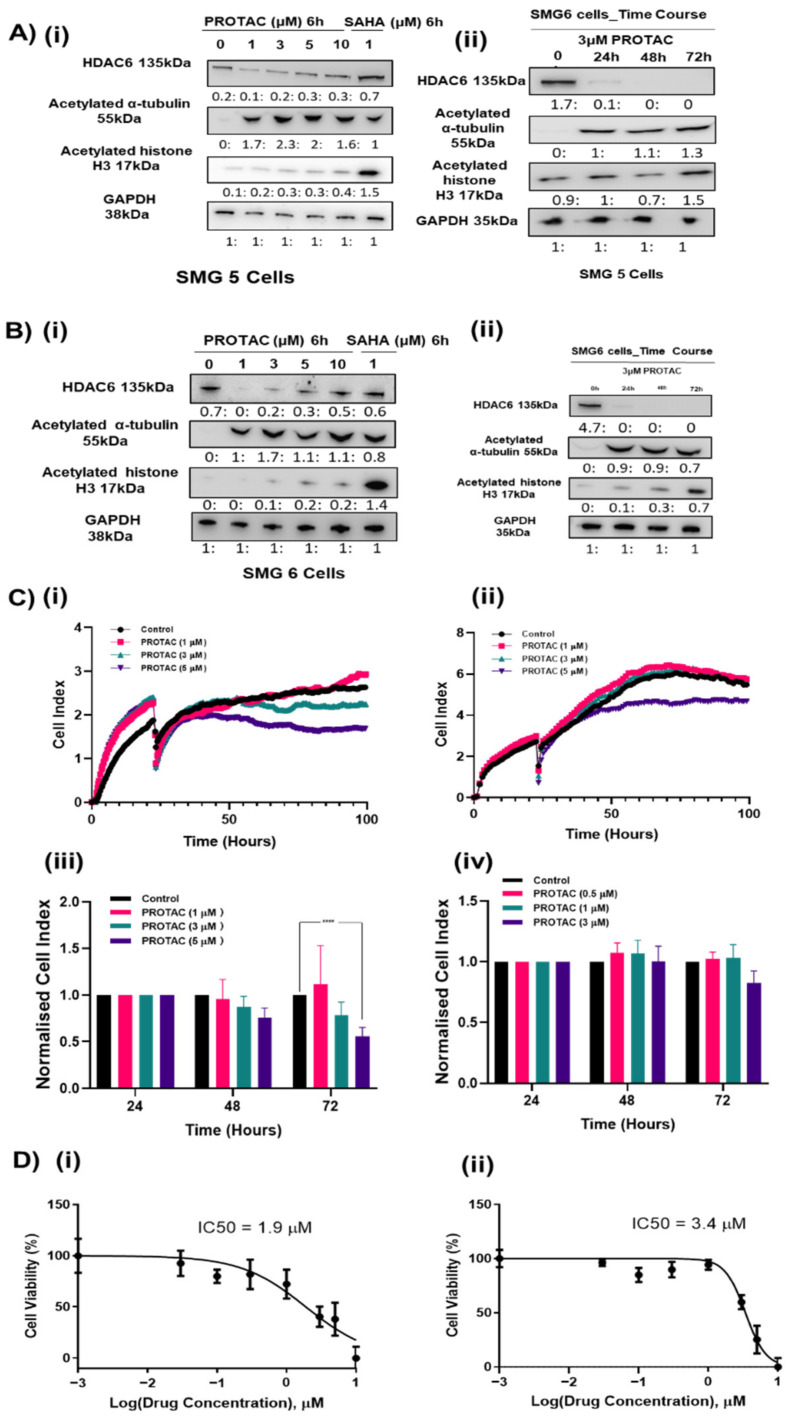
Efficacy of PROTAC treatment on ovarian cancer cell growth. (**A**) SMG 5 cells were treated by PROTAC for 6 h. SAHA, a pan inhibitor of HDACs, was used as a positive control. Degradation efficacy and selectivity of PROTAC was tested by western blot. GAPDH was used as a loading control. Degradation efficacy of 3 μM PROTAC was tested over time. Experiments performed at least *n* = 2. Uncropped Western Blots are available in Figures S10 and S11 (**B**) SMG 6 cells were treated by PROTAC for 6 h. Degradation efficacy and selectivity of PROTAC was tested by western blot. GAPDH was used as a loading control. Degradation efficacy of 3 μM PROTAC was tested over time. Experiments performed at least *n* = 2. Uncropped Western Blots are available in Figures S12 and S13. (**C**)/(**i)**,(**ii**) Representative graphs from the real time xCELLigence proliferation system comparing growth profiles of SMG cells following treatment with PROTAC. (**C**)/(**iii)**,(**iv**) Cell index values were normalized to both 24 h and control, observing the rate of proliferation (24–96 h). Wells were analyzed in duplicate, and three biological replicates were performed to validate results. Experiments were analyzed by a two-way ANOVA. **** *p* < 0.0001. Error bars represent SEM. (**C**)/(**i)**,(**iii**) SMG5, (**C**)/(**ii)**,(**iv**) SMG6. (**D**) Effect on cell viability after treated by PROTAC for 48 h was determined by MTT assay (*n* = 3).

The high expression of HDAC6 in HGSOC tumors and disruption of cell proliferation and migration in HDAC6 siRNA-treated cells suggest that HDAC6 may provide a survival advantage to HGSOC. Therefore, we investigated the pharmacological inhibition of HDAC6 as a potential targeted therapy to treat HGSOC. ACY-1215 is a selective HDAC6 inhibitor currently being assessed in phase I and phase II clinical trials. ACY-1215 has an IC50 of 5 nM and has demonstrated selective HDAC6 inhibition up to ~1 μM [22]. At higher concentrations, in vitro activity assays demonstrated that ACY-1215 has some activities against HDAC4, HDAC5, HDAC7, HDAC9, HDAC11, Sirtuin1, and Sirtuin2 and against HDAC8 at an IC50 = 0.1 μM [22]. Results from this current study showed that ACY-1215 produced a potent inhibition of HDAC6 at 0.1 µM in SMG5 and 0.5 µM in SMG6, which was measured by a robust accumulation of its primary substrate, acetylated α-tubulin, up to 24 h (Figure 4A). While higher doses also showed a high accumulation of acetylated α-tubulin, levels of acetylated histone 3, a nuclear HDAC substrate, showed increased acetylation when using >5 μM of ACY-1215 in SMG5 cells and >3 µM ACY-1215 in SMG6 cells, indicating other HDACs may be inhibited at higher concentrations (Figure 4A). We assessed real time growth in response to ACY-1215 in the four SMG cell lines. A dose-dependent decrease in cell proliferation was observed (Figure 4B and Figure S4B). However, selective concentrations of ACY1215 (<3 µM) moderately slowed the growth of the SMG5 and SMG6 cells. Selective concentrations of ACY-1215 appear to be cytostatic and only when non-selective ACY-1215 concentrations (10 μM) were used was a clear decrease in cell viability observed (Figure 4C). We also examined the effects of targeted degradation of HDAC6 with a proteolysis targeting chimera (PROTAC) molecule. The HDAC6 targeting PROTAC produced a robust decrease in HDAC6 expression at 100 nM with a dose dependent increase in acetylated α-tubulin without affecting the acetylation levels of histone H3 up to 10 µM (Figure 5A(i),B(i)). Cell viability of SMG5 and SMG6 cells was affected with lower concentrations of PROTAC than that of ACY-1215 (IC 50 = 1.9 µM and 3.4 µM, respectively) (Figure 5D). However, the growth rate of the cells was not significantly affected at these concentrations (Figure 5C). Cellular resistance to PROTAC molecules can develop following long-term treatment as reported by several studies [38,39]. Decreased cell viability with this HDAC6 targeting PROTAC was previously shown to result from targeted degradation of IKAROS family zinc finger (IKZF) proteins at micromolar doses, although we did not measure any significant expression of IKZF1 and IKZF2 in ovarian cancers (Figure S5). SMG6s were less sensitive to HDAC6 degradation consistent with both HDAC6 siRNA and ACY1215 data, indicating that this cell line may be less dependent on HDAC6 functions.

HDAC6 knockdown produced a robust inhibition of HGSOC migration, in which we next examined the impact of pharmacological HDAC6 inhibition on the migratory properties of ovarian cancer cells in a trans-well setting. Selective HDAC6 inhibition using ACY-1215 at >1 μM lowered the migration of HGSOC cells (Figure 4D). Again, the concentration of ACY-1215 required to lower HGSOC migration far exceeded doses that produced biochemical inhibition of α-tubulin acetylation, in which the higher non-selective concentrations were required to inhibit cell migration comparable to HDAC6 knockdown experiments.

### 2.5. HDAC6 Inhibition Did not Affect Responses to Paclitaxel in HGSOC Cells

**Figure 6:**
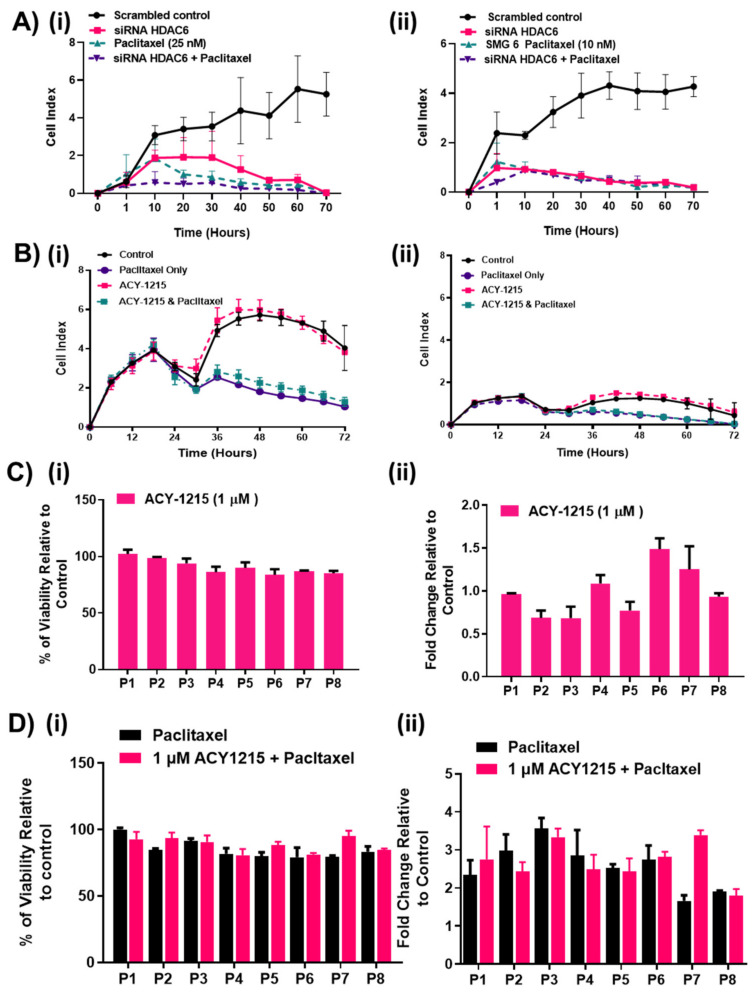
HDAC6 inhibition effect on paclitaxel responses. (**A**) SMG 5 (**i**) and SMG 6 (**ii**) cells were transfected with HDAC6 siRNA followed by treatment with IC50 doses of paclitaxel for further 72 h (*n* = 2). (**B**) SMG 5 (**i**) and SMG 6 (**ii**) cells were pre-treated with 1 μM ACY-1215 and then treated with 1 μM ACY-1215 in combination with paclitaxel for a further 72 h. Real-time detection of cellular impendence was measured to calculate normalized cell index utilizing the xCELLigence System. Experiments were analyzed by a two-way ANOVA. Error bars represent SEM. (**C**,**D**) Primary cell cultures from eight HGSOC patients were pretreated with 1 µM ACY-1215 or a combination of 1 µM ACY-1215 and paclitaxel for 48 h. (**C**)/(**i)**,(**ii**) Viability was measured using the Promega CellTiter-Fluor™ Cell Viability Assay, while (**D**)/(**i)**,(**ii**) caspase activity as an indicator of apoptosis was measured using the Promega Caspase-Glo^®^ 3/7 Assay. Experiments were analyzed by a two-way ANOVA. (*n* = 2). Error bars represent SEM.

The actions of paclitaxel and HDAC6 compete to modulate the post-translation modification of α-tubulin with paclitaxel promoting α-tubulin acetylation and HDAC6 decreasing it. Therefore, it has been proposed that high HDAC6 expression could attenuate paclitaxel responses in cells. In addition, upon microtubule stabilization by Taxol, structural changes in both β- and α-tubulin occur, the modification of α-tubulin by ACY-1215 could structurally alter β tubulin and potentially affect paclitaxel binding [40]. Therefore, we tested the effects of HDAC6 knockdown and the pharmacological inhibition on paclitaxel responses in the SMG cell lines. In SMG5 and SMG6 cell lines, neither HDAC6 siRNA nor selective HDAC6 inhibition with 1 μM ACY1215 altered the dynamics of the paclitaxel responses measured by xCELLigence (Figure 6A,B). Comparing the paclitaxel treated arm of each experiment, no difference in the level of the cell index or rate of decline of the cell index were recorded for HDAC6 inhibition, suggesting that selective HDAC6 inhibition did not affect paclitaxel responses in SMG cells. To further investigate the results obtained, primary cell cultures from eight HGSOC patients were pre-treated with 1 μM ACY-1215 for 6 h to lower HDAC6 levels (Figure 6C) and further treated with a combination of 1 μM ACY-1215 with or without paclitaxel for 48 h (Figure 6D). Both cell viability and caspase 3/7 activity were measured using the Promega CellTiter™ Cell Viability Assay and Promega Caspase-Glo^®^ 3/7 Assay (Promega, Southampton, UK), respectively. At selective concentrations, ACY-1215 failed to cause any significant changes in cell viability or apoptosis of the primary cultures consistent with earlier observations, in which selective ACY-1215 drug concentrations only modestly slowed the growth of the SMG cell lines. The P6 and P7 primary cultures recorded 0.5-fold increase in apoptosis (Figure 6Cii), suggesting perhaps a partial response in these patient’s culture. Again, for most primary cultures, no differences in caspase 3/7 activity between paclitaxel and paclitaxel combined with 1 μM ACY-1215 was measured (Figure 6D). Primary culture P7 was the only culture sensitized to paclitaxel in combination with HDAC6 inhibition. Overall, this data suggests that HDAC6 inhibition may not improve responses to paclitaxel. Conversely, HDAC6 inhibition did not inhibit paclitaxel responses in the cell lines and primary cultures tested, which suggests that HDAC6 inhibition may not interfere with paclitaxel treatment regimens.

## 3. Discussion

Ovarian cancer is treated by surgery aimed at debulking the tumor to remove macroscopic disease followed by a taxane and platinum-based chemotherapy regimen. This approach prolongs survival but the majority of patients relapse owing to chemo-resistance [41]. If we are to improve patient survival, HGSOC needs new treatment options. In this research paper, we set out to evaluate the contribution of HDAC6 to the survival of HGSOC cells in which we show the association between low HDAC6 and an increased risk of death in patients presenting with HGSOC. In contrast, we show that lowering HDAC6 levels inhibited HGSOC cell spreading, proliferation, and migration. The limitations of the current study include low patient numbers and lack of existing studies in HGSOC to compare with. While Yano et al., 2018 [23], demonstrated an association between low nuclear HDAC6 expression and poorer survival of all ovarian cancer subtypes, when they assessed HDAC6 expression of each of the ovarian cancer subtypes, they did not find any association with HDAC6 protein expression in HGSOC [23]. We suggest that the HDAC6 expression in HGSOC may have dichotomized the HGSOC samples into molecularly distinct subtypes where low HDAC6 associated with hard-to-treat cancers and high HDAC6 with more treatable cancers. Indeed, we have previously shown that the miR-433 microRNA, which is associated with poorer outcomes in HGSOC, senescence and chemo-resistance, regulates HDAC6 [42,43]. In this study, we showed that the induction of senescence leads to the down regulation of HDAC6 expression (Figure S1B), in which decreased HDAC6 expression has also been linked to replicative senescence [44]. Therefore, low HDAC6 expressing tumors may be representative of this phenotype while high HDAC6 expressing HGSOC cells may have evolved a survival dependency on HDAC6. The heat shock protein 90 (HSP90)/HDAC6 chaperone machinery, is considered a major determinant of mutant p53 stabilization [45] and HDAC6 inhibition has shown preferential sensitivity in p53 mutant cancers [46,47]. As HGSOC are mostly p53 mutated tumors, we hypothesized that HDAC6 inhibition could be a treatment option for HGSOC.

From the comparison of responses from three independent methods of downregulating the activity of HDAC6, we have revealed that the loss of HDAC6 activity in cells slows the growth and migration of HGSOC cells in which downregulating HDAC6 expression with siRNA or a PROTAC was more potent than catalytic inhibition with ACY-1215. The differences in responses obtained by eliminating HDAC6 expression compared with enzymatic inhibition could arise because HDAC6 possess functions that are independent of its catalytic activity. Specifically, the scaffold functions of HDAC6 would only be affected by eliminating its expression [44]. Ustinova et al. [44] have proposed a model of HDAC6 binding to microtubules via ionic interactions with a newly discovered microtubule binding domain [45]. In this model, HDAC6 was capable of binding to microtubules in the presence of the HDAC6 inhibitor, Nexturastat A. Furthermore, the loss of HDAC6 deacetylation on tubulin could be overcome by the presence of other members of the HDAC family, such as Sirtuin 2 (SIRT2) and HDAC8 [46,47]. Therefore, cells could readily compensate for HDAC6 catalytic inhibition and may explain the lack of efficacy reported for current HDAC6 enzymatic inhibitors. To overcome limitations of poorly selective enzymatic inhibitors, PROTACs, which are novel degrading agents provide targeted degradation of cellular proteins. The PROTAC used in this study, is highly selective for HDAC6 without affecting other HDACs even at higher concentrations. Moreover, several HDAC6 targeting PROTACs also produced anti-proliferative effects in various cancer cells [48,49,50]. Thus, downregulating HDAC6 expression may have greater anti-cancer effects compared to enzymatic inhibition with small molecule inhibitors.

Depetter et al.,2019 [30] present data demonstrating that HDAC6 inhibition yields modest anti-cancer responses that may be easily overcome by resistance mechanisms as discussed [30]. The anti-cancer effects of ACY-1215 is most frequently reported to result from the synergistic effect of HDAC6 inhibition with other drugs, such as inhibition with proteosomal pathway inhibitors [40]. Recently, Moufarrij et al., 2020 [51] showed that combing epigenetic modulators, such as DNA methyltransferase inhibitors and HDAC6i, increases anti-tumor immune signaling from cancer cells and has beneficial effects on the ovarian tumor immune microenvironment [52]. ACY-1215 is a relatively innocuous orally available drug which permits the administration of more cytotoxic compounds, such as bortezimib, at tolerable concentrations [53]. The co-administration of HDAC6 inhibitory therapy with paclitaxel has been previously explored as a synergistic combination, although much of the existing data reported non-selective HDAC6 inhibition [30]. We did not measure improved responses in primary cultures of HGSOC cells co-treated with paclitaxel and ACY-1215, suggesting that the co-administration of ACY-1215 at selective concentrations may not improve the cytotoxic responses of HGSOC cells to paclitaxel. The results of an abbreviated Phase Ib study of Ricolinostat (ACY-1215) and recurrent ovarian cancer demonstrated preclinical efficacy in preventing and/or reversing chemotherapy-induced peripheral neuropathy [49] that may permit the administration of paclitaxel at more effective concentrations in affected patients. Therefore, improved clinical responses to the combination of HDAC6i and paclitaxel may occur independent of tumor cell death. Of note, the phase 1b study also reported synergistic anti-tumor responses with various chemotherapies, suggesting a molecular bias of certain tumors for HDAC6 inhibition which should be assessed further to determine if improved tumor microenvironment responses or direct cytotoxicity of the tumor cells are responsible. For example, mutations of the ARID1A gene is associated with increased sensitivity to HDAC6 inhibition in clear cell carcinomas [54]. HGSOC is not usually associated with this mutation suggesting another molecular bias may exist in high HDAC6 expressing HGSOC. An investigation of the molecular characteristics of HDAC6 expressing HGSOC tumors could improve the application of HDAC6i therapy in the treatment of HGSOC.

## 4. Materials and Methods

### 4.1. Patients, Tissue Specimens, and Histopathological Review

HGSOC specimens (46) were obtained from consenting women by laparotomy at St. James’s Hospital, Dublin, Ireland. Ethical approval was given by St. James’s Hospital and the Adelaide Meath and National Children’s Hospital research ethics committee (biobank approval 21 December 2004, 041213/12904 and subsequent amendments in 13 August 2009 (Ref 2009/29/01) and 24 February 2014 (Ref. 2014-10 List 11)). All included patients provided written informed consent for International Federation of Gynecology and Obstetrics (FIGO)’s guidelines participation (www.figo.org). Tumors were staged according to the. All women received adjuvant Carboplatin/paclitaxel chemotherapy, with charts monitored retrospectively and follow-up information, including age, optimum surgical debulking, and time to recurrence, recorded. Overall Survival (OS) was calculated from the date of surgery to the date of death. Table 1 shows the clinicopathological features of the 46 cases included in this study.

### 4.2. HDAC6 Immunohistochemistry (IHC) and Manual Scoring

Formalin-fixed paraffin embedded (FFPE) sections (5 μm) were stained using a HDAC6 (1:50) polyclonal antibody (Abcam, Cambridge UK), previously optimized [55,56] and performed on an automated platform (Bond^TM^system, Leica MicroSystems^TM^, Newcastle, UK), as previously described [57]. HDAC6 staining was analyzed in the HGSOC cohort by two independent pathologists. Scoring was performed based on the intensity of staining and graded as follows: negative 0, low +1 (less than 10% of viable tumor stained, intermediate +2 (10–50% of viable tumor stained) and high +3 (more than 50% of viable tumor stained). Discrepancies between scores were re-evaluated jointly by a third pathologist (EK), and a consensus was reached.

### 4.3. HDAC6 mRNA Meta-Analysis

For the ovarian survival hazard ratio forest plot, hazard ratios, confidence intervals, and *p*-values were was obtained for each of the risk groups from “SurvExpress” [58] (Table S1). Data was not found in two of the SurvExpress ovarian databases, GSE19161 and GSE30009. The forest plot was then created using the R-statistics package (version R-3.5.2).

### 4.4. Cell Line Culture

SV40 immortalized SMG cell lines were established by the Stratified Medicine Group in Queens University Belfast (QUB). Cell lines established include (1) SMG 5: Metastatic ovarian carcinoma/grade 4, (2) SMG 6: High grade serous papillary/grade 3C, (3) SMG 19: High-grade serous ovarian carcinoma/grade 3, and (4) SMG 35: metastatic high-grade serous carcinoma/grade 4. All established cell lines were grown in DMEM/F12, GlutaMAX media supplemented with 10% fetal calf serum (FCS) (Gibco, Paisley, UK), 100 IU/mL penicillin, 100 μg/mL streptomycin, 2 mM glutamine, and 1 mM sodium pyruvate and maintained as monolayers, incubated at 37 °C, under 5% CO_2_. Eight primary cell cultures from eight different tumors of resected from patients with HGSOC, were grown in Imperial College, London, UK. Cells were subsequently cultured in RPMI 1640 medium supplemented with 20% FCS, 20 mM L-glutamine and 1% penicillin/streptomycin and 1 mM sodium pyruvate (Gibco). Eight patient-derived HGSOC cell lines were provided by Ovarian Cancer Action Research Center, Imperial College London, UK and cultured according to [59].

### 4.5. SDS-PAGE and Western Blotting

SDS-PAGE and western blotting were performed as described previously [58] with the following antibodies; acetylated α-tubulin, acetylated H3, (Cell Signaling, London, UK), WT-1, CK8, p53 (Abcam, Cambridge UK), HDAC6 (Merck, Hertfordshire, UK). β-actin and GAPDH (Sigma, Hertfordshire, UK) were used as loading controls. The levels of protein expression were assessed using a SuperSignal™ West Pico PLUS Chemiluminescent detection kit (ThermoFisher, Paisley, UK), and G:BOX imaging system (Syngene, Cambridge, UK).

### 4.6. Transient Transfection and Treatments

Double-stranded small interference RNA (siRNA) for HDAC6, and the silencer-negative siRNA as a control, were purchased from Ambion, Aberdeenshire, UK. Cells were transfected with siRNA (250 nM) using lipofectamine RNAiMAX (Thermofisher, Paisley, UK) as per manufacturer’s protocol. Cells were then collected or re-seeded for further investigation, 48 h after transfection. The induction of p21 and p16 in HT1080 cells was achieved by treating the cells with 50 μM IPTG (Isopropyl β-D-1-thiogalactopyranoside) (Fisher Scientific, Paisley, UK) over three days.

### 4.7. xCELLigence,

Real-time proliferation and migration assays were performed using the xCELLigence RTCA DP instrument (ACEA Biosciences, Cheshire, UK), which was placed in a humidified incubator at 37 °C and 5% CO_2_. Cells were seeded into E-plates 16 (ACEA Biosciences, Cheshire, UK), according to the manufacturer’s instructions and left overnight to adhere. For cell proliferation experiments with ACY-1215 (Selleckchem, Cambridgeshire, UK), the drug was added following 24 h adherence. The impedance value in electron flow (resistance to an alternating current) of each well was monitored by the xCELLigence system and reported as a cell index value.

Real-time monitoring of cell trans-well migration, was carried out using a xCELLigence machine according to the manufacturer’s guidelines. Each treatment condition was performed in duplicate and the electrical impedance was measured every 5 min for 24 h by RTCA software. The trans-well migration was expressed as the cell index, which is a measure for the change in impedance at each time point.

### 4.8. Immunocytochemistry

SMG cells were fixed in 4% Paraformaldehyde for 30 min at room temperature and blocked for 1 h in PBS containing 1% bovine serum albumin (BSA, Sigma Aldrich, Hertfordshire, UK) and 0.01% Tween 20 (Sigma Aldrich). Next, the cells were incubated for 1 h at room temperature with the epithelial marker CK8 primary antibody (Abcam). After washing in PBS, the cells were incubated in appropriate secondary antibody (Anti-rabbit and Alexa Flour 568 IgG (Abcam, UK) for 1 h at room temperature and observed under a fluorescence microscope (EVOS FL, Life Technologies, Paisley, UK).

### 4.9. Quantitative Reverse-Transcription PCR (RT-PCR)

Total RNA was extracted from the SMG cell lines using TRIzol Reagent (Thermo Fisher Scientific). RNA concentration and purity were checked with a NanoDrop spectrophotometer (Thermo Fisher Scientific). Complementary DNA (cDNA) was synthesized using High-Capacity RNA-to-cDNA Kit (Life Technologies). Quantitative real time polymerase chain reaction (qRT-PCR) was performed with Power SYBR^®^ Green PCR Mastermix (Thermo Scientific) in 10 μL reactions containing 2 μL of 1:5 cDNA dilution and 0.5 μM of gene-specific primers for 40 cycles in a LightCycler 480 (Roche, Hertfordshire, UK). Oligonucleotide primers for each gene are presented in Table S1. β-actin was used as an endogenous control.

### 4.10. Drug Sensitivity Assay

The CellTiter 96 Proliferation Assay (MTS) (Promega, Southampton, UK) was used to determine the chemosensitivity of the cell lines, while the CellTiter-Fluor Cell Viability Assay (Promega, Southampton, UK) was used in all primary cells. Response curves were generated for cisplatin and paclitaxel (Sigma). Graphpad prism was used to fit a dose response curve and to calculate the 50% growth inhibition values (IC_50_) with error and 95% confidence intervals.

### 4.11. Caspase 3/7 Assay

Caspase 3/7 activity was measured using a Promega Caspase-Glo^®^ 3/7 assay (Promega, Southampton, UK) as per the manufacturer’s instructions. Luminescence reading was measured to determine caspase activation on the FLUOstar Omega microplate reader (BMG LABTECH, Bucks, UK).

### 4.12. Statistical Analysis

Data are presented as the mean ± SEM for at least 3 independent experiments unless otherwise stated in the text and figure legends. The student’s *t*-test was conducted using Prism 5 (Graphpad Software version 5.03) to compare the means. R/Bioconductor (“Survival” package 2.37–4) was used to generate Kaplan-Meier survival curves with significance determined by using a Log-Rank test. For univariate/multivariate survival analyses, the Cox proportional hazard model was employed (R version 3.01). Multivariate analysis was built using a reverse step-wise regression. Statistical significance was calculated at a 95% confidence level. Two-way ANOVA and Sidak’s multiple comparison test were conducted using Prism 8 to compare the difference of cell index between groups.

## 5. Conclusions

In conclusion, our results indicate that the over-expression of HDAC6 is a favorable prognostic marker in the HGSOC ovarian cancer subtype and highlights HDAC6 as a potential therapeutic target for HGSOC. We strongly demonstrated how selective inhibition of the deacetylase activity of HDAC6 with ACY-1215 has limited activity as a monotherapy and suggest that lowering HDAC6 enzymatic activity may be insufficient to eliminate HDAC6 cell functions. While targeting HDAC6 may be a valuable therapeutic target for treating HGSOC, an evaluation of the functional specificity of HDAC6 inhibition should be further explored in HGSOC cells so that HDAC6i therapy could be targeted to responsive molecular subtypes of HGSOC.

## Figures and Tables

**Table 1:** Clinical characteristics of ovarian cancer cases included in this study.

Clinical Characteristic	Low HDAC6	High HDAC6	Overall
	*n* = 23 (%)	*n* = 23 (%)	*n* = 46 (%)
Age (years)			
Mean	54	60	57
Median	54	61	59
Standard Deviation	9	10	10
Range	41–74	36–76	36–76
FIGO Stage			
I/II	1 (4.3%)	5 (27.7%)	6 (13%)
III/IV	22 (95.7%)	18 (78.3%)	40 (87%)
Grade			
Unknown	1 (4.3%)	1 (4.3%)	2 (4.3%)
Low	1 (4.3%)	0 (0%)	1 (2.2%)
Intermediate	8 (34.9%)	6 (26.1%)	14 (30.5%)
High	13 (56.5%)	16 (69.6%)	29 (63%)
Cytoreduction/Cytoreductive surgery			
Unknown	2 (8.7%)	0 (0%)	2 (4.3%)
sub-Optimal > 1	3 (13%)	7 (30.4%)	10 (21.8%)
Optimal ≤ 1	18 (78.3%)	16 (69.6%)	34 (73.9%)

## Supplementary Materials

The following are available online at https://www.mdpi.com/2072-6694/12/12/3734/s1, Table S1: Ovarian survival hazard ratios, confidence intervals and *p*-values for HDAC6 mRNA expression, Figure S1: HDAC6 levels in ovarian cancer cell lines. p21 and p16 inducible models of cellular senescence were used to study the correlation of senescence with HDAC6, Figure S2: Paclitaxel and Cisplatin treatment for 48 h of treatment. Cell viability was measured using MTS assay, Figure S3: SiRNA II knockdown of HDAC6 lower the proliferation of SMG cells,: High concentration of HDAC6 inhibitor affected cell proliferation, Figure S5: Comparing the mRNA expression level of IKZF1&3 and HDAC6 in ovarian cancer cells, Figure S6: Uncropped Western Blot of Figure 2B, Figure S7: Uncropped Western Blot of Figure 2D, Figure S8: Uncropped Western Blot of Figure 4A(i), Figure S9: Uncropped Western Blot of Figure 4A(ii), Figure S10: Uncropped Western Blot of Figure 5A(i), Figure S11: Uncropped Western Blot of Figure 5A(ii), Figure S12: Uncropped Western Blot of Figure 5B(i), Figure S13: Uncropped Western Blot of Figure 5B(ii).
