# Supplementary material for: HDAC6 Degradation Inhibits the Growth of High-Grade Serous Ovarian Cancer Cells
Source: Cancers (Basel). 2020 Dec 11;12(12):3734. doi: 10.3390/cancers12123734 (PMC7762972; doi:10.3390/cancers12123734)
Supplement: Supplementary file 1 [file cancers-12-03734-s001.pdf]

Supplementary Material: HDAC6 Degradation Inhibits the Growth of High-Grade Serous Ovarian Cancer Cells

Ahlam Ali, Fengyu Zhang, Aaron Maguire, Tara Byrne, Karolina Weiner-Gorzel, Stephen Bridgett, Sharon O'Toole, John O'Leary, Caitlin Beggan, Patricia Fitzpatrick, Amanda McCann and Fiona Furlong

Table S1. Ovarian survival hazard ratios, confidence intervals, and *p*-values for HDAC6 mRNA expression.

Study No.	Sample No.	HR	CIHR_Low	CIHR_High	*p* HR	Study
1	285	1.02	0.7	1.49	0.92	GSE9891
2	255	1	0.7	1.43	1.00	GSE32062
3	63	1.26	0.68	2.34	0.46	GSE18520
4	415	1.13	0.78	1.64	0.51	GSE13876
6	80	1.51	0.9	2.56	0.12	GSE14764
7	153	1.6	0.91	2.82	0.10	GSE3149
8	195	1.13	0.8	1.59	0.49	GSE26712
9	44	1.77	0.63	4.99	0.28	GSE8842
10	28	1.08	0.44	2.62	0.87	GSE19829, GPL570
11	110	0.96	0.59	1.58	0.89	GSE17260
12	784	1.12	0.92	1.36	0.25	Ovarian Meta-base
13	70	1.11	0.49	2.52	0.81	GSE19829-GPL8300
15	58	1.64	0.83	3.26	0.16	GSE30161
16	40	1.75	0.74	4.14	0.20	GSE32063
17	578	1.02	0.82	1.28	0.84	TCGA
18	58	0.62	0.27	1.41	0.25	GSE31245
19	28	16.74	2	139.9	0.01	GSE23554
20	247	0.98	0.7	1.35	0.88	OV-TCGA (June 2016)
21	82	2.01	1.2	3.36	0.01	OV-AU, ICGC (June 2016)

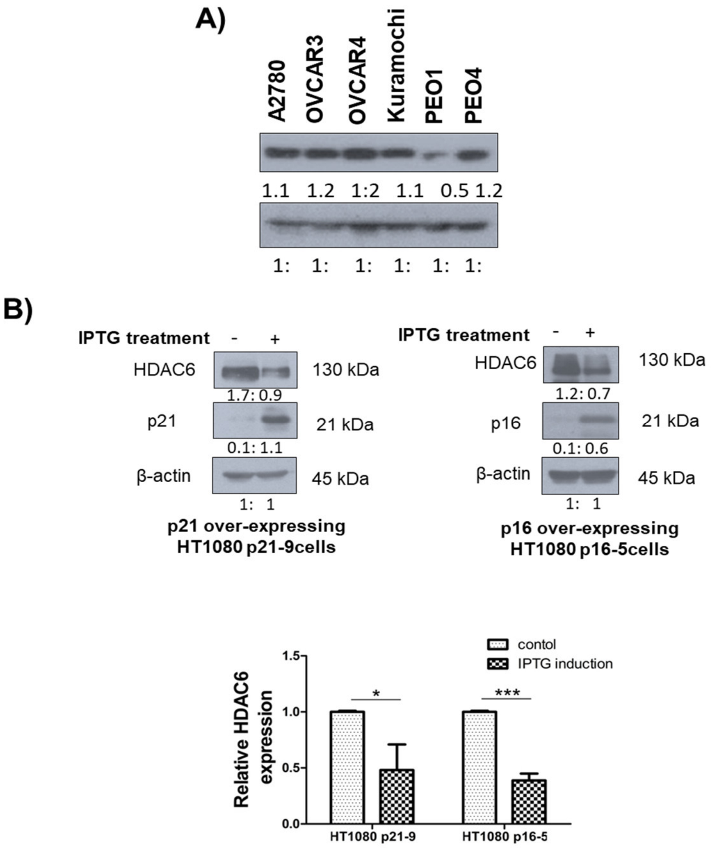

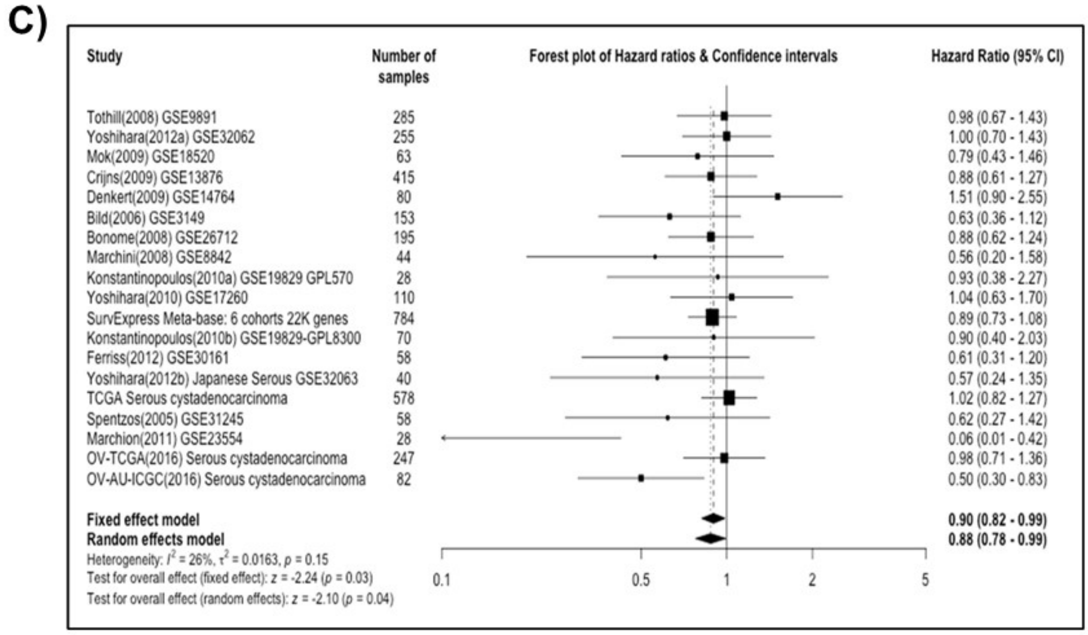

Figure S1. (**A**) HDAC6 levels in ovarian cancer cell lines. Representative images of western blots showing HDAC6 expression. $n = 3$. (**B**) p21 and p16 inducible models of cellular senescence were used to study the correlation of senescence with HDAC6. HT1080 fibrosarcoma cell line model, either p21 or p16 was induced by the treatment with Isopropyl β-D-1-thiogalactopyranoside (IPTG) for 72 h. qRT-PCR analysis of mRNA demonstrated a significant downregulation of HDAC6 in the p21-inducible model ($p < 0.001$ and $p = 0.017$) and in the p16-inducible model ($p < 0.001$ and $p < 0.001$) respectively. Experiment was performed at least $n = 3$. Error bars represent SEM. (**C**) Meta-analysis of highest vs. lowest category of HDAC6 gene expression levels and risk to overall cancer survival.

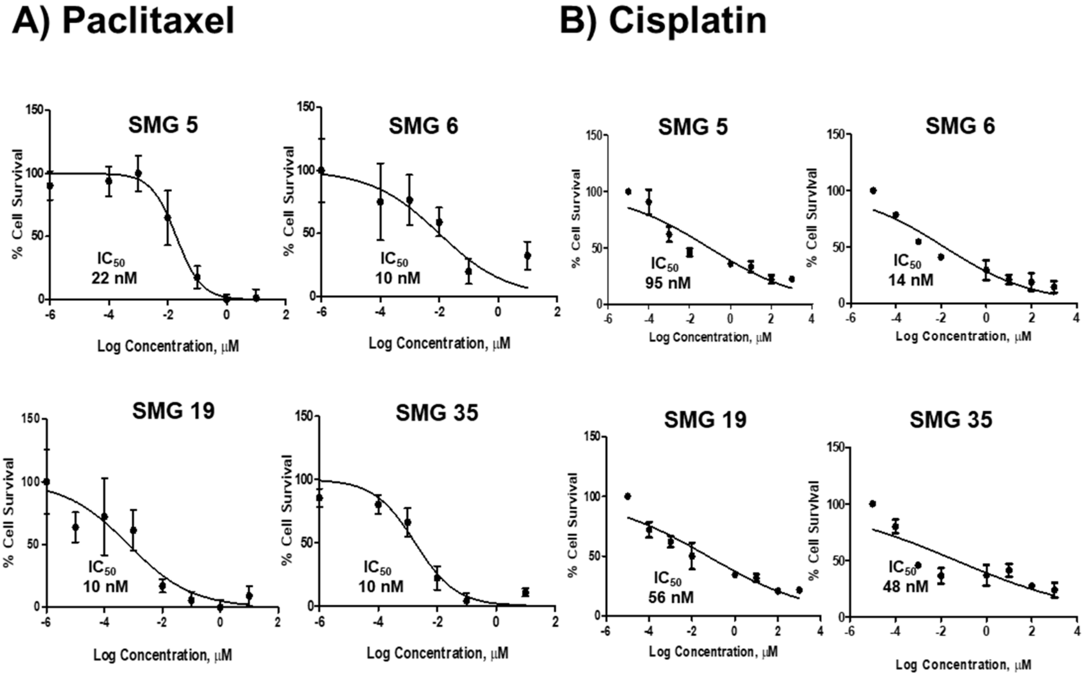

Figure S2. (**A**)Paclitaxel and (**B**) Cisplatin treatment for 48 hours of treatment. Cell viability was measured using MTS assay. Experiment was performed at least $n = 3$. Error bars represent SEM.

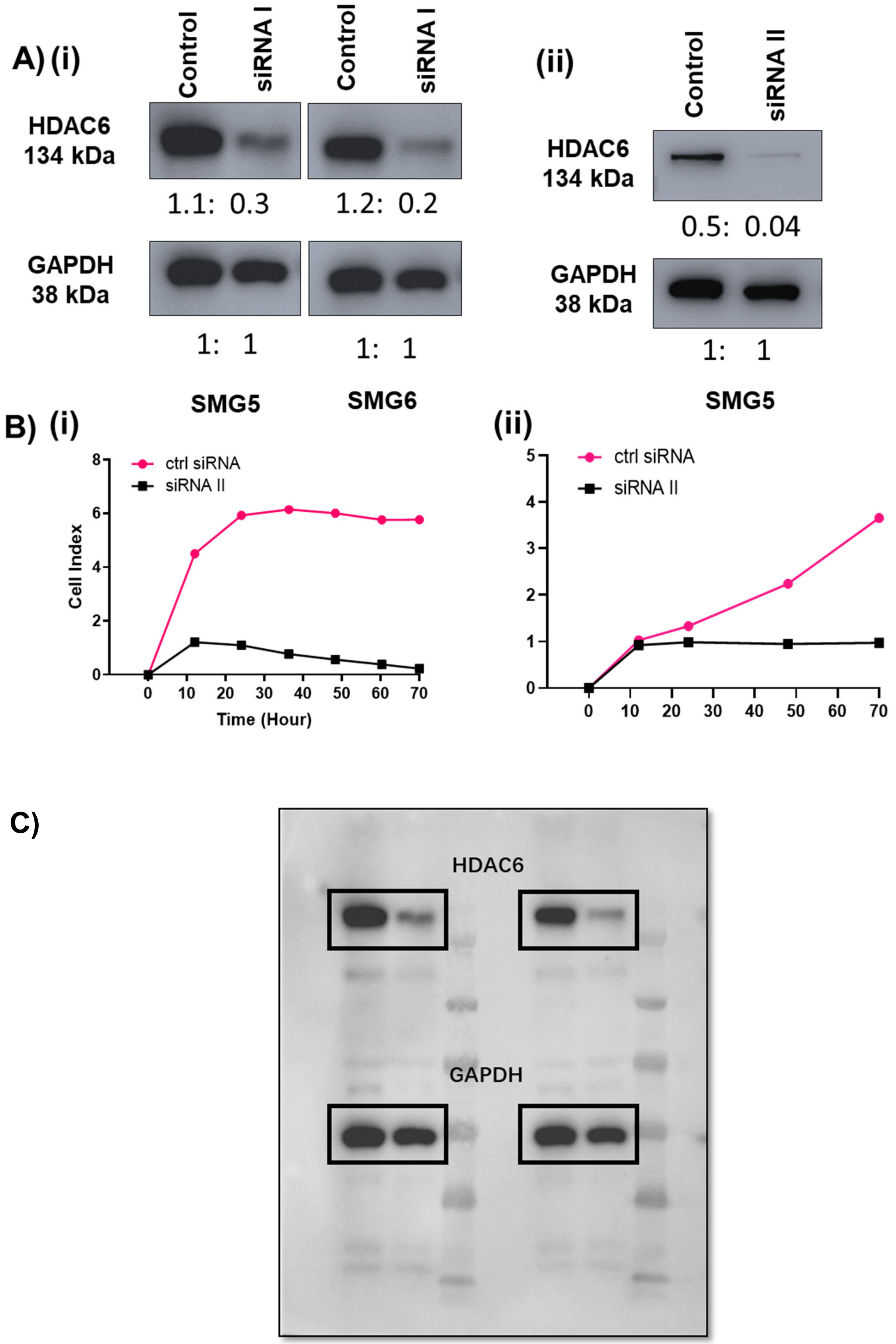
A) (i)
Control
siRNA I
Control
siRNA I
HDAC6
134 kDa
1.1: 0.3
1.2: 0.2
GAPDH
38 kDa
1: 1
1: 1
SMG5
SMG6
(ii)
Control
siRNA II
HDAC6
134 kDa
0.5: 0.04
GAPDH
38 kDa
1: 1
SMG5
B) (i)
ctrl siRNA
siRNA II
Cell Index
Time (Hour)
(ii)
ctrl siRNA
siRNA II
C)
HDAC6
GAPDH

D)

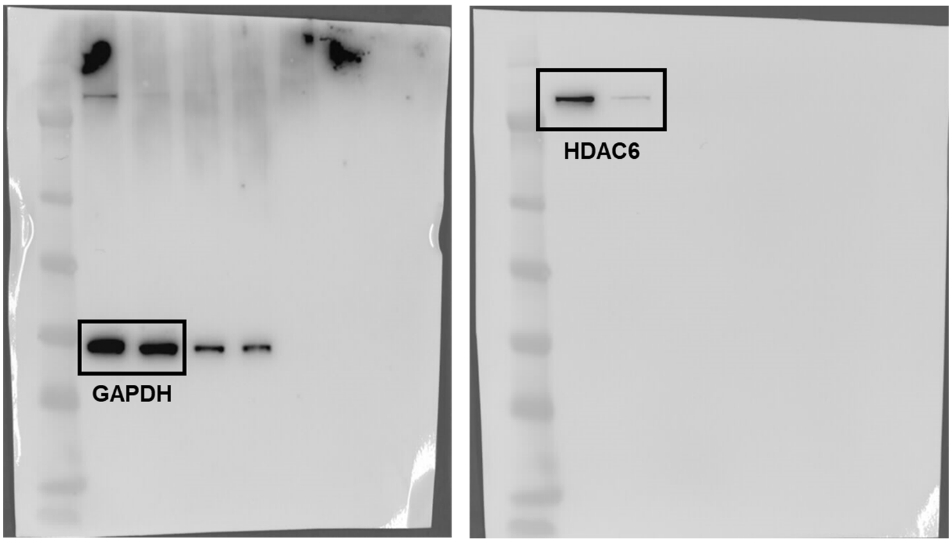

Figure S3. siRNA II knockdown of HDAC6 lower the proliferation of SMG cells. (**A**) (**i**) SMG cells were transfected with HDAC6 siRNA I or negative scrambled control and knockdown down was confirmed with immunoblot. (**ii**) SMG cells were transfected with HDAC6 siRNA II or negative scrambled control and knockdown down was confirmed with immunoblot. Uncropped WB are available in Figure S16 and S17.(**B**) Cell proliferation assay of SMG 5 (**i**) and SMG 6 (**ii**) following HDAC6 transfection. Real-time detection of cellular impendence was measured to calculate normalised cell index with the xCELLigence System. All experiments performed at least $n = 2$. (C) Uncropped Western Blots of (**A**)(**i**) and (**D**) Uncropped Western Blots of (**A**)(**ii**).

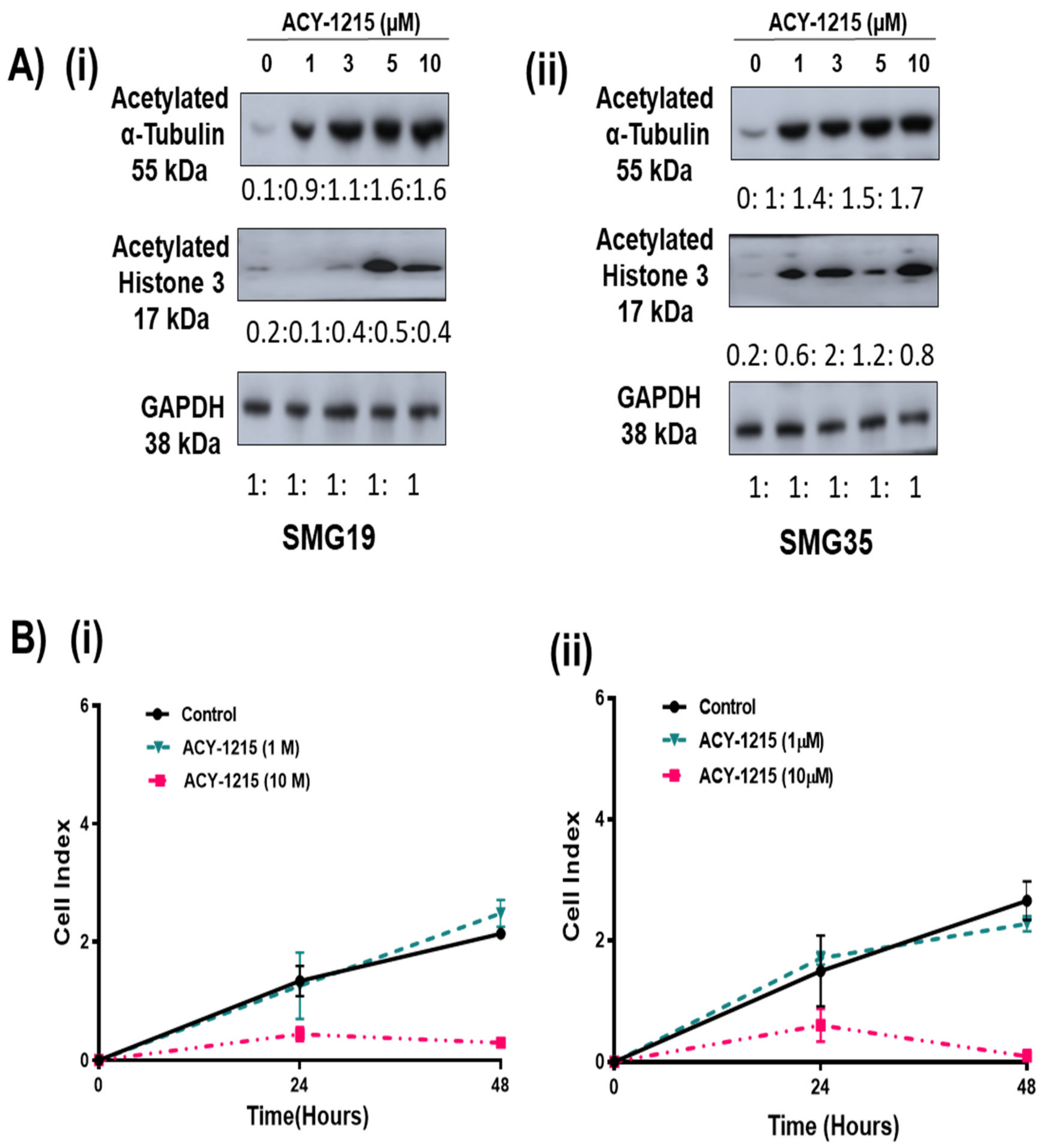
A) (i)
ACY-1215 (µM)
0 1 3 5 10
Acetylated α-Tubulin 55 kDa
0.1:0.9:1.1:1.6:1.6
Acetylated Histone 3 17 kDa
0.2:0.1:0.4:0.5:0.4
GAPDH 38 kDa
1: 1: 1: 1: 1
SMG19
(ii)
ACY-1215 (µM)
0 1 3 5 10
Acetylated α-Tubulin 55 kDa
0: 1: 1.4: 1.5: 1.7
Acetylated Histone 3 17 kDa
0.2: 0.6: 2: 1.2: 0.8
GAPDH 38 kDa
1: 1: 1: 1: 1
SMG35
B) (i)
Control
ACY-1215 (1 M)
ACY-1215 (10 M)
Cell Index
Time(Hours)
(ii)
Control
ACY-1215 (1µM)
ACY-1215 (10µM)
Cell Index
Time (Hours)

C)

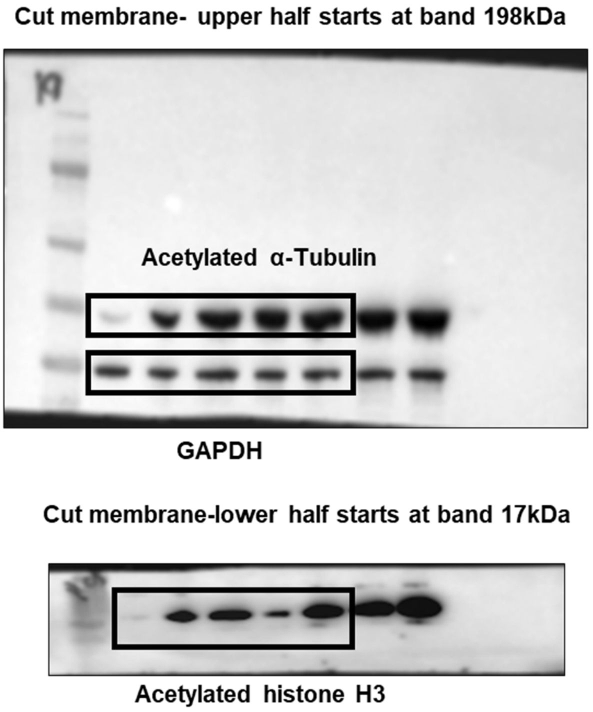

D)

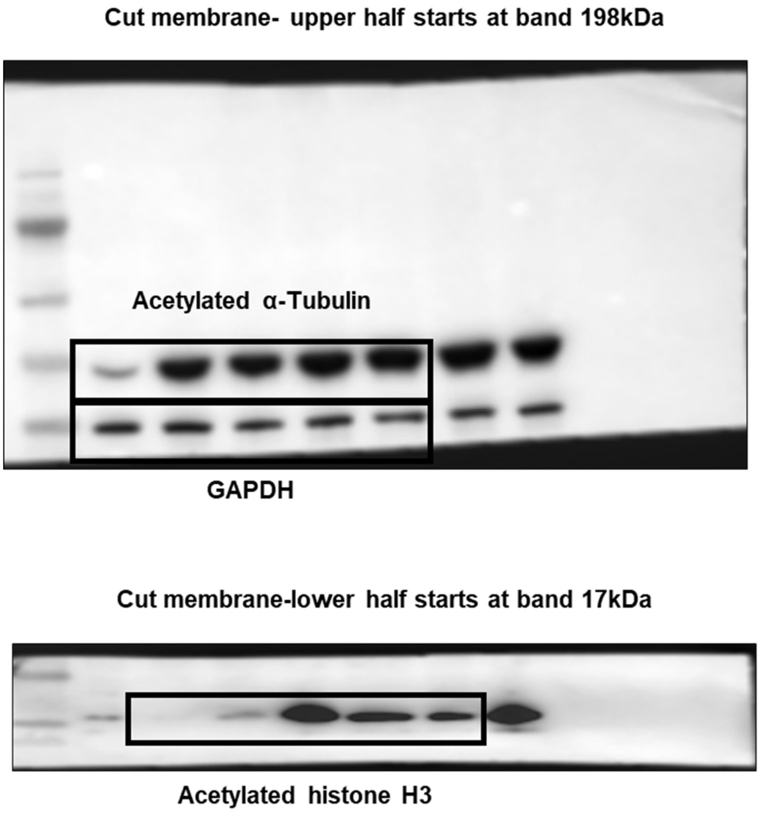

Figure S4. High concentration of HDAC6 inhibitor affected cell proliferation. (**A**) SMG19 (**i**) and SMG35 (**ii**) cells treated with 1–10 μM of ACY-1215 and α-tubulin and H3 levels were measured by western blot analysis. GAPDH was used as a loading control. Experiment performed $n = 1$. Uncropped WB are available in Figure S18 and Figure S19. (**B**) Cell proliferation were measured using xCELLigence system after treating SMG19 (**i**)(**iii**) and SMG35 (**ii**)(**iv**) with 1 μM and 10 μM ACY-1215.

Experiments were performed n = 2 and analysed by a two-way ANOVA. Error bars represent SEM. (**C**) Uncropped Western Blots of (**A**)(**i**) and (**D**) Uncropped Western Blots of (**A**)(**ii**).

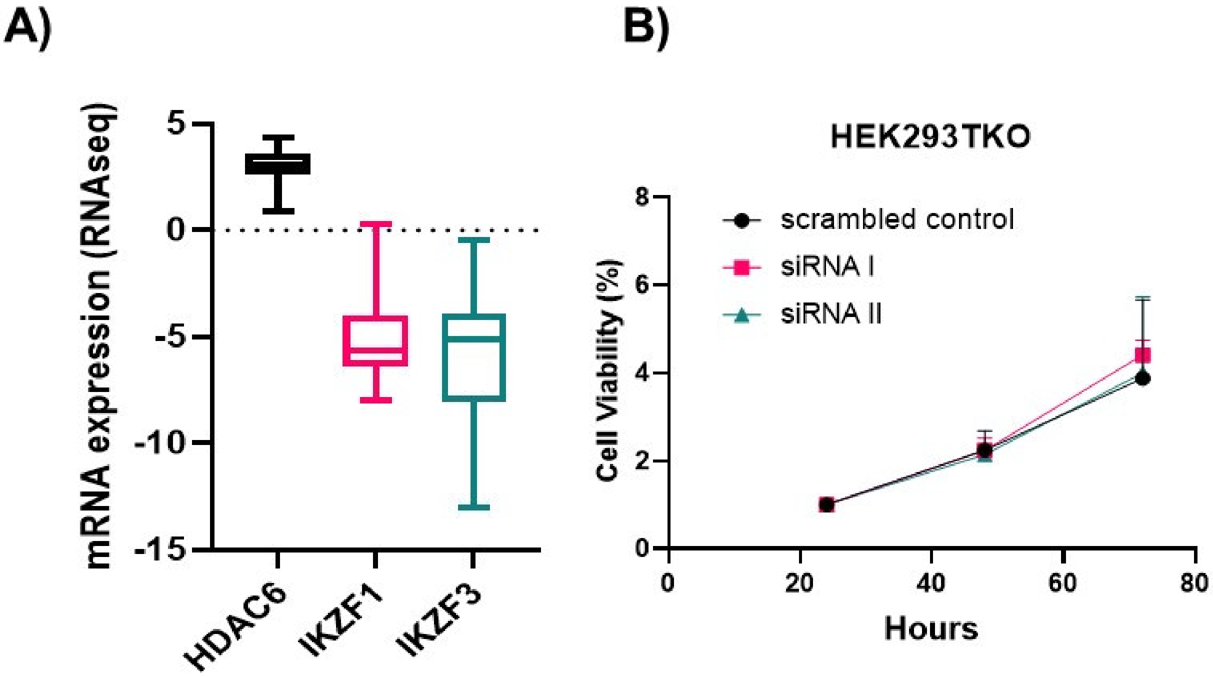

Figure S5. (**A**) Comparing the mRNA expression level of IKZF1&3 and HDAC6 in ovarian cancer cells. The data was obtained from Cancer Cell Line Encyclopedia (CCLE) (**B**) Stable cell line HEK293T HDAC6 knockdown cells were transfected with HDAC6 siRNA I, HDAC6 siRNA II, and negative scrambled control. Cell viability assays were performed. Experiments were performed n = 3. Error bars represent SEM**.**

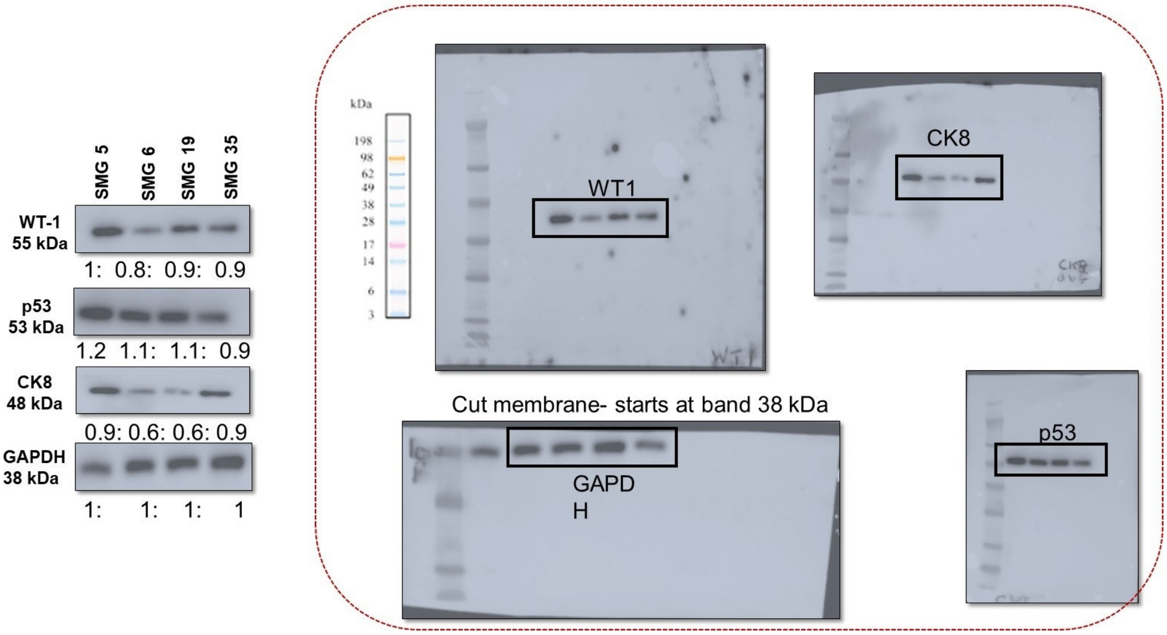

Figure S6. Uncropped Western Blot of Figure 2B.

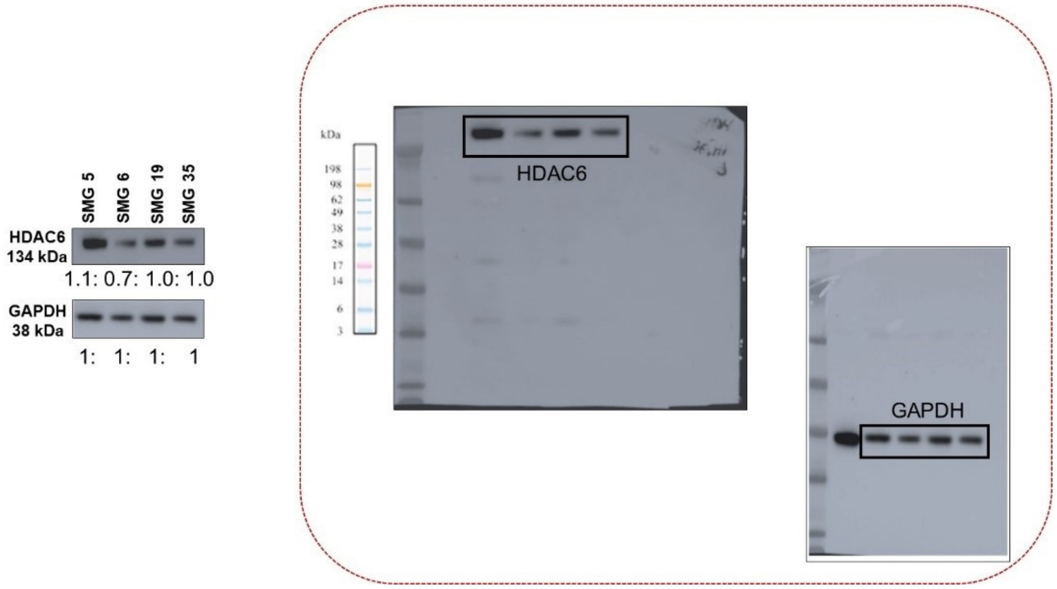

Figure S7. Uncropped Western Blot of Figure 2D.

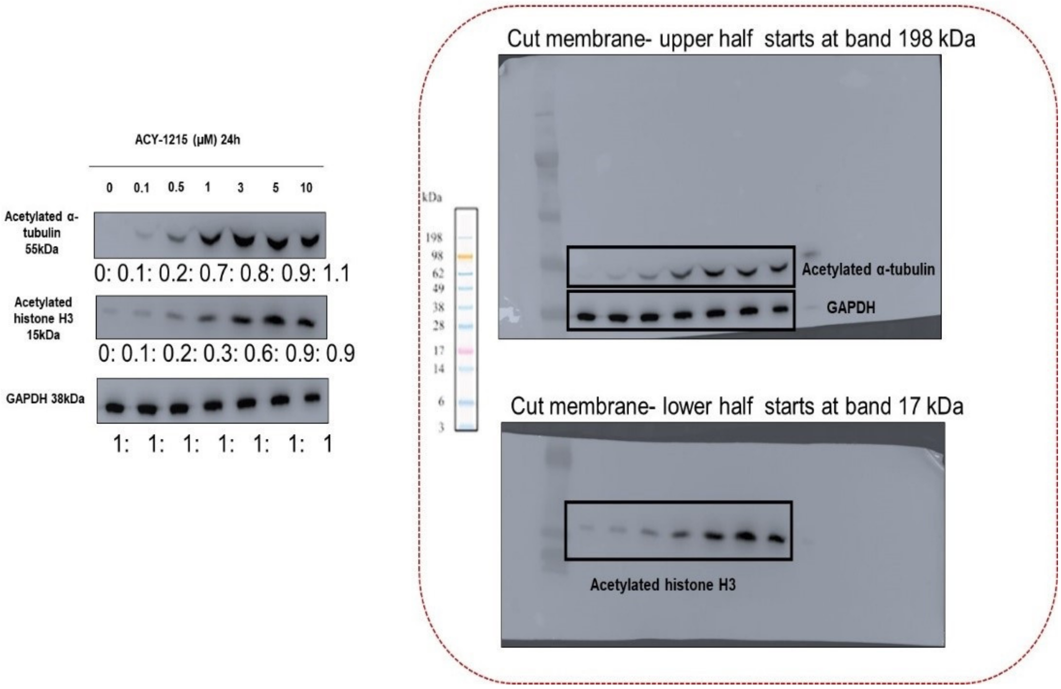

Figure S8. Uncropped Western Blot of Figure 4A(i).

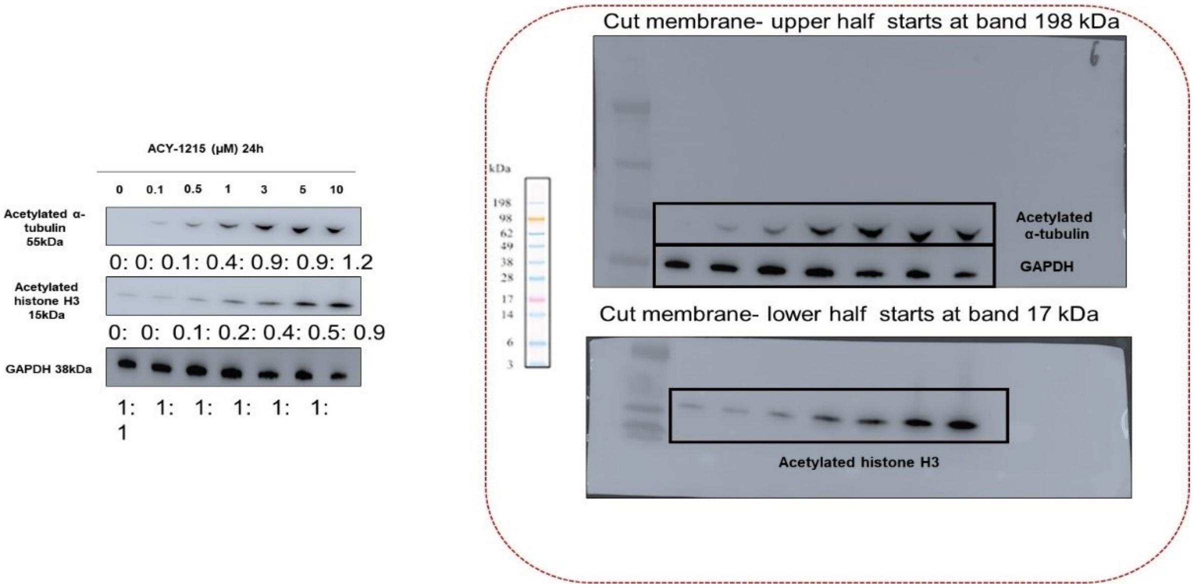

Figure S9. Uncropped Western Blot of Figure 4A(ii).

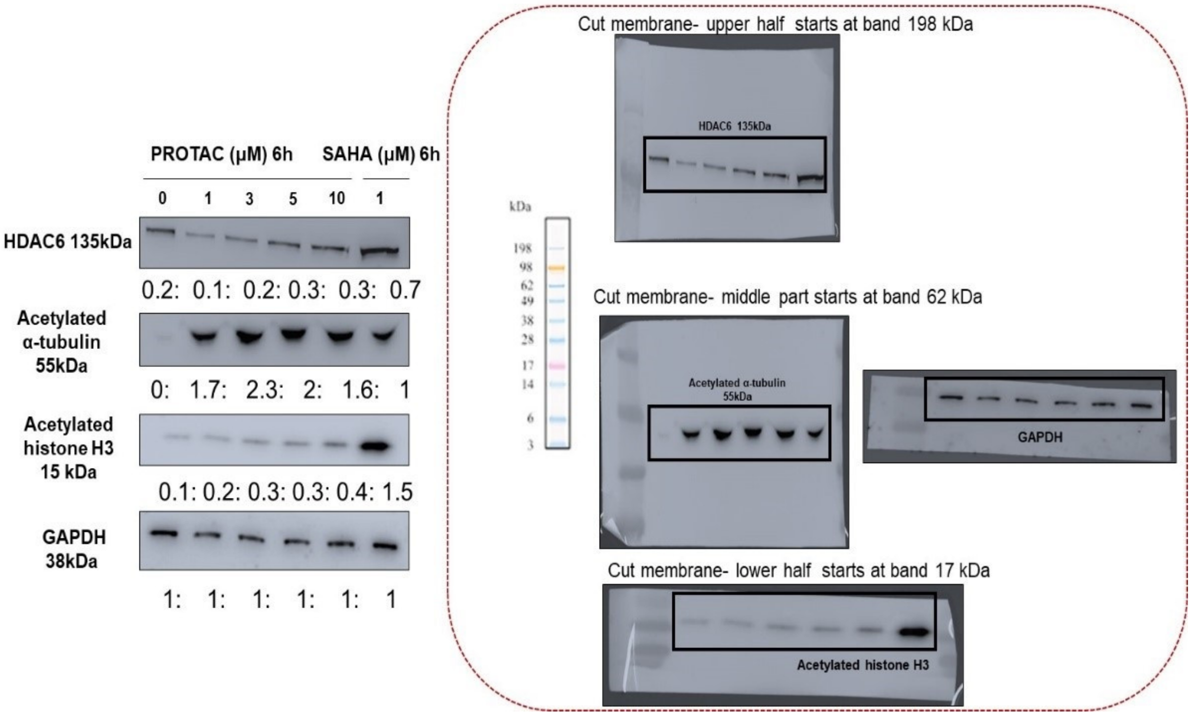

Figure S10. Uncropped Western Blot of Figure 5A(i).

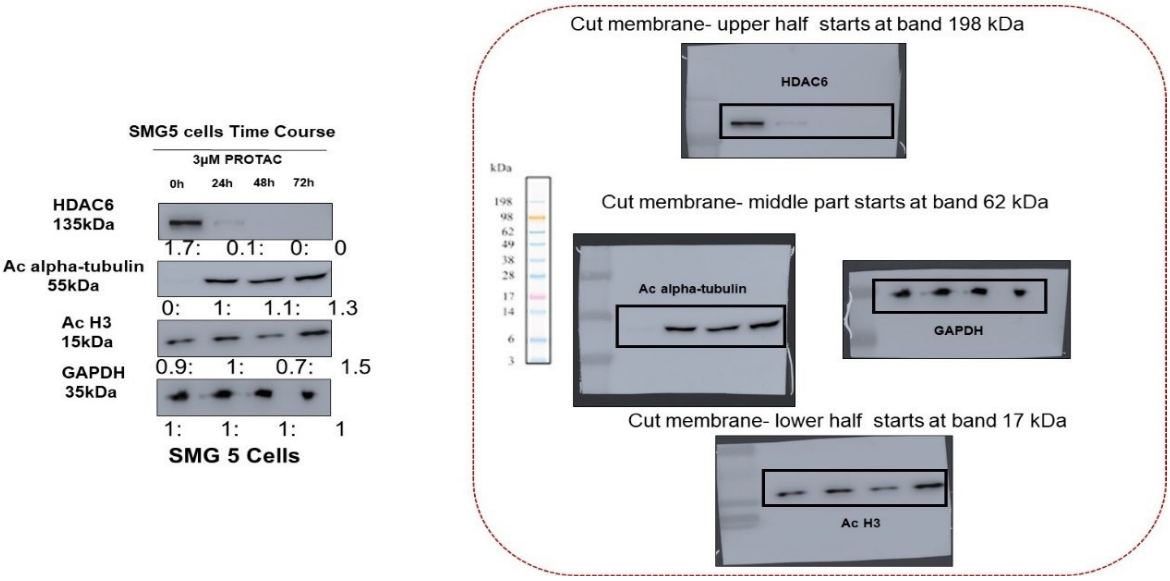

Figure S11. Uncropped Western Blot of Figure 5A(ii).

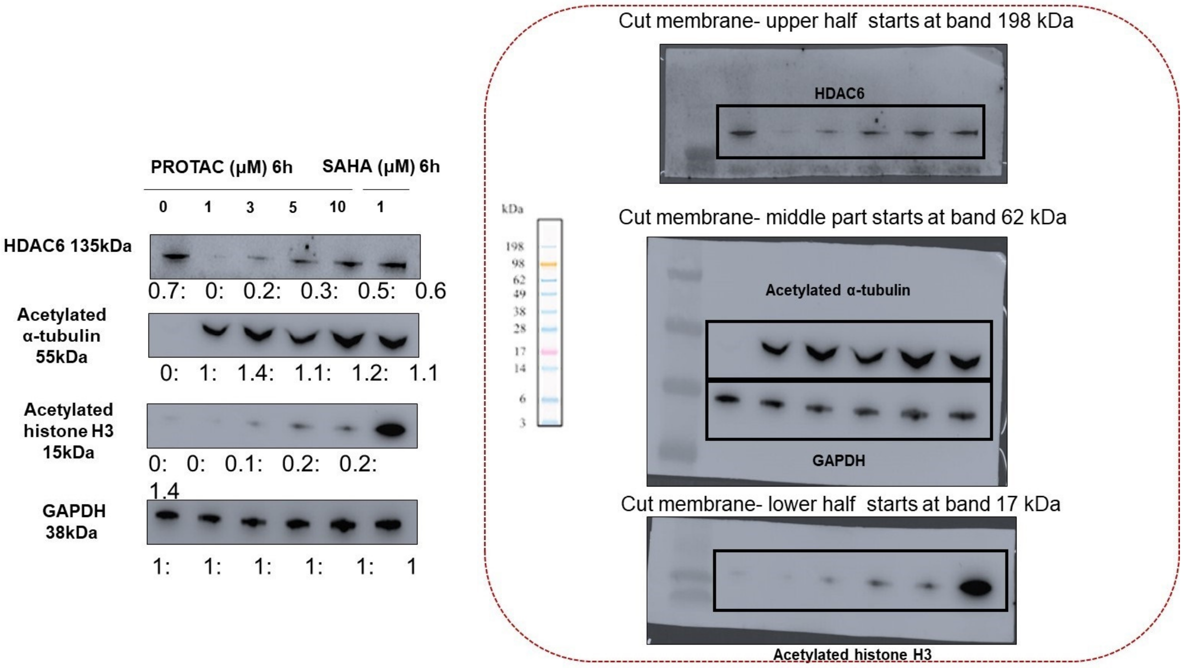

Figure S12. Uncropped Western Blot of Figure 5B(i).

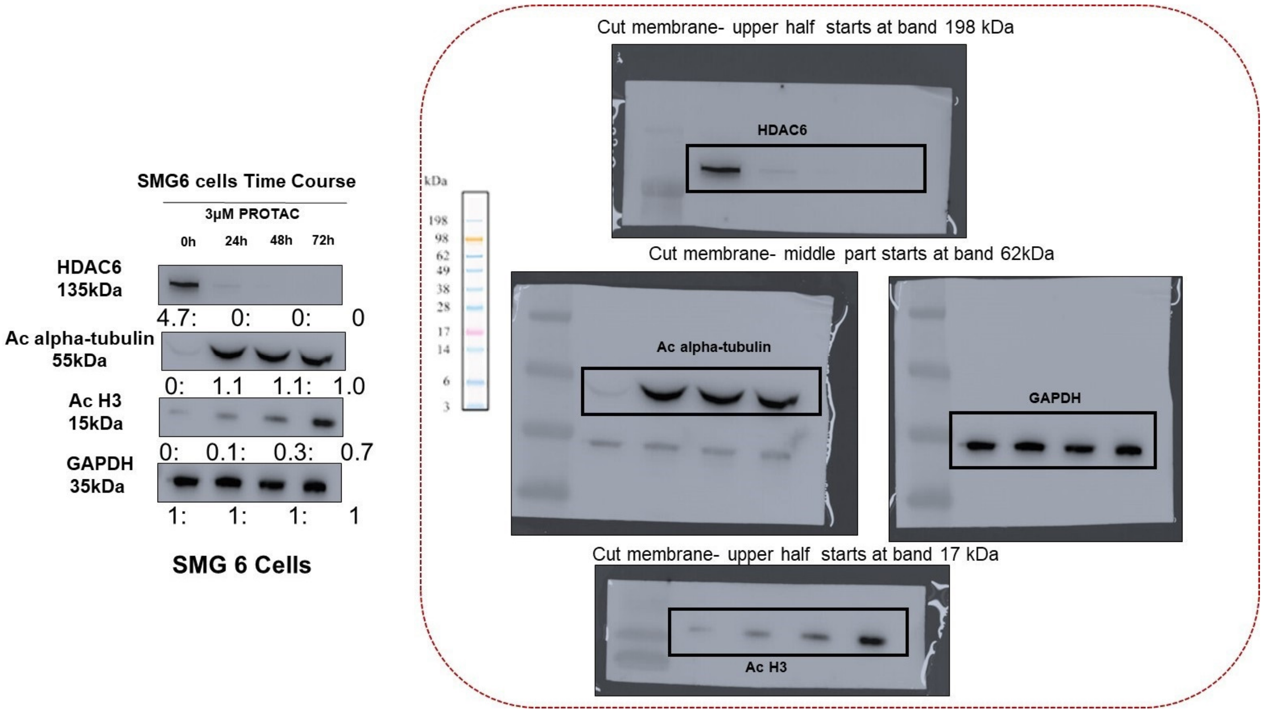

Figure S13. Uncropped Western Blot of Figure 5B(ii).

Publisher's Note: MDPI stays neutral with regard to jurisdictional claims in published maps and institutional affiliations.

© 2020 by the authors. Licensee MDPI, Basel, Switzerland. This article is an open access article distributed under the terms and conditions of the Creative Commons Attribution (CC BY) license (http://creativecommons.org/licenses/by/4.0/).
